# Unique diagnostic signatures of concussion in the saliva of male athletes: the Study of Concussion in Rugby Union through MicroRNAs (SCRUM)

**DOI:** 10.1136/bjsports-2020-103274

**Published:** 2021-03-23

**Authors:** Valentina Di Pietro, Patrick O'Halloran, Callum N Watson, Ghazala Begum, Animesh Acharjee, Kamal M Yakoub, Conor Bentley, David J Davies, Paolo Iliceto, Gabriella Candilera, David K Menon, Matthew J Cross, Keith A Stokes, Simon PT Kemp, Antonio Belli

**Affiliations:** 1 University of Birmingham, Institute of Inflammation and Ageing, Birmingham, UK; 2 NIHR Surgical Reconstruction and Microbiology Research Centre, University Hospitals Birmingham NHS Foundation Trust, Birmingham, UK; 3 Marker Diagnostics UK Limited, the BioHub, Birmingham research park, Birmingham, UK; 4 College of Medical and Dental Sciences, Institute of Cancer and Genomic Sciences, Centre for Computational Biology, University of Birmingham, Birmingham, UK; 5 Institute of Translational Medicine, University Hospitals Birmingham NHS, Foundation Trust, Birmingham, UK; 6 S&P Statistics and Psychometrics Ltd, Rome, Italy; 7 Division of Anaesthesia, University of Cambridge, Cambridge, UK; 8 Department for Health, University of Bath, Bath, UK; 9 Premier Rugby Limited, Twickenham, London, UK; 10 Rugby Football Union, Twickenham, London, UK; 11 Faculty of Epidemiology and Public Health, London School of Hygiene and Tropical Medicine, London, UK

**Keywords:** trauma, concussion, brain, contact sports, diagnosis

## Abstract

**Objective:**

To investigate the role of salivary small non-coding RNAs (sncRNAs) in the diagnosis of sport-related concussion.

**Methods:**

Saliva was obtained from male professional players in the top two tiers of England’s elite rugby union competition across two seasons (2017–2019). Samples were collected preseason from 1028 players, and during standardised head injury assessments (HIAs) at three time points (in-game, post-game, and 36–48 hours post-game) from 156 of these. Samples were also collected from controls (102 uninjured players and 66 players sustaining a musculoskeletal injury). Diagnostic sncRNAs were identified with next generation sequencing and validated using quantitative PCR in 702 samples. A predictive logistic regression model was built on 2017–2018 data (training dataset) and prospectively validated the following season (test dataset).

**Results:**

The HIA process confirmed concussion in 106 players (HIA+) and excluded this in 50 (HIA−). 32 sncRNAs were significantly differentially expressed across these two groups, with let-7f-5p showing the highest area under the curve (AUC) at 36–48 hours. Additionally, a combined panel of 14 sncRNAs (let-7a-5p, miR-143-3p, miR-103a-3p, miR-34b-3p, RNU6-7, RNU6-45, Snora57, snoU13.120, tRNA18Arg-CCT, U6-168, U6-428, U6-1249, Uco22cjg1, YRNA_255) could differentiate concussed subjects from all other groups, including players who were HIA− and controls, immediately after the game (AUC 0.91, 95% CI 0.81 to 1) and 36–48 hours later (AUC 0.94, 95% CI 0.86 to 1). When prospectively tested, the panel confirmed high predictive accuracy (AUC 0.96, 95% CI 0.92 to 1 post-game and AUC 0.93, 95% CI 0.86 to 1 at 36–48 hours).

**Conclusions:**

SCRUM, a large prospective observational study of non-invasive concussion biomarkers, has identified unique signatures of concussion in saliva of male athletes diagnosed with concussion.

## Introduction

Sport-related concussion is defined as ‘a traumatic brain injury induced by biomechanical forces that typically results in the rapid onset of short-lived impairment of neurological function that resolves spontaneously^›^.^1^ A high percentage of cases may go misdiagnosed or unidentified and concerns have emerged about the long-term brain health of athletes exposed to repeated concussions.

The extremely poor objective diagnostic tests after an index event has the potential to expose individuals to the risk of further single or multiple concussive events before the initial concussion has resolved. Conventional neuroimaging (CT and MRI scanning) is normal by definition, and the diagnosis currently relies on a clinician’s interpretation of the observed signs, symptoms reported and cognitive/neuropsychometric and/or physical evaluations (eg, balance or oculo-vestibular assessments).^2 3^ The assessments are not specific for concussion and require subject honesty and cooperation, operator training and prescriptive test conditions. The short-term consequences of a missed diagnosis range from a prolonged recovery period, often with protracted and pervasive symptoms, to a heightened risk of further injuries, including rarely, catastrophic brain swelling (second impact syndrome).^4 5^

In recent years, there has been focus on the development and validation of objective diagnostic tools for concussion, both within traditional clinical settings and pitch side at sporting events. Several blood biomarkers have been intensively studied, including S100β, glial fibrillar acidic protein (GFAP), ubiquitin carboxy-terminal hydrolase L1 (UCH-L1), neuron-specific enolase (NSE), Tau, neurofilament light protein (NFL) and beta-amyloid protein.^6–10^

A blood assay using GFAP and UCH-L1 has Food and Drug Administration approval to evaluate the requirement for a CT scan and rule out haemorrhagic pathology in traumatic brain injury (TBI). However, diagnosis of concussion expands beyond the exclusion of pathology visible on CT imaging, as the majority of injuries will not result in structural abnormalities identifiable with standard imaging methods.^11–13^

Recently, rapid advances in high-throughput technologies, such as next generation sequencing (NGS), have allowed investigation of new classes of molecules, such as RNA species, as potential biomarkers. Among these, microRNAs (miRNAs, miRs), which belong to the small non-coding RNAs (sncRNAs) (20–200 nucleotides in length), are the most studied, with evidence of a miRNA signature that varies according to TBI severity in blood, cerebrospinal fluid (CSF) and saliva.^14–24^ However, other classes of sncRNAs such as small nuclear RNA (snRNA), small nucleolar RNA (snoRNA), transfer RNA (tRNA), YRNA and piwiRNA (piRNA) have emerged as new candidate biomarkers in several pathologies. The role of the majority of these molecules is not fully understood, but it is evident that 90% of our genome encoding for untranslated RNA has functional activity in normal biology and in pathological conditions.^25–27^

In professional male rugby union in England, clinicians’ evaluations of athletes who have sustained a head injury with the potential to result in concussion are supported by a standardised head injury assessment (HIA) protocol,^28^ providing an excellent setting in which to examine biomarkers of concussion and clinical outcomes.

We assessed sncRNAs as potential salivary biomarkers of sport-related concussion in professional male rugby players, with the following objectives

- *Biomarkers identification:* To discover whether any sncRNAs are differentially expressed in players with a clinical diagnosis of concussion compared with other groups.
- *Longitudinal analysis:* To evaluate the time course of the sncRNA expression response in players with a clinical diagnosis of concussion.
- *Biomarkers validation and predictive model:* To determine whether a combination of sncRNAs can predict the outcome of a structured professional clinical assessment.

## Methods

### The SCRUM study

#### Ethics

The Study of Concussion in Rugby Union through MicroRNAs (SCRUM) is a prospective, observational cohort study in which cases and controls are compared, and is part of the REpetitive COncussion in Sport (RECOS) research programme.^29^ The study was carried out in the highest two tiers of senior male professional domestic rugby union in England in the 2017–2018 and 2018–2019 seasons. The study was authorised by the University of Birmingham research ethics committee and by the East of England NHS ethics committee (ref. 11-0429AP28). All participants gave written informed consent in accordance with the Declaration of Helsinki. The study was preregistered with the International Standard Randomised Controlled Trials Number in February 2018 (ISRCTN16974791), it followed the Strengthening the Reporting of Observational Studies in Epidemiology (STROBE) reporting guideline, and the methodology, including the analysis plan, was published in 2018.^30^

#### Participants

**Figure 1:**
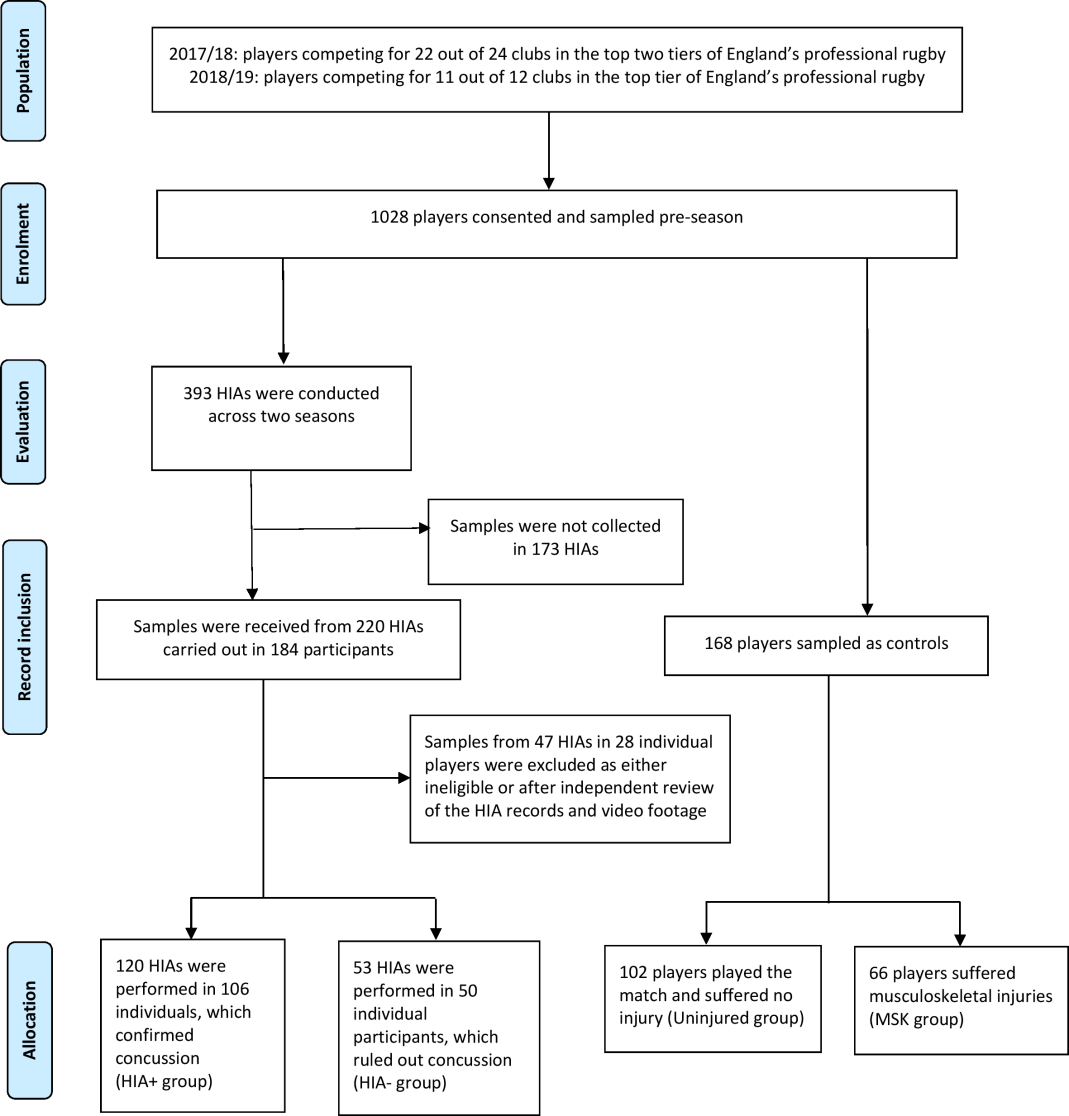
Study profile. Participants were divided into concussion confirmed (HIA+) or ruled out (HIA−) after their head injury assessment (HIA), or controls, represented by players who played in the same game but were uninjured or had had a musculoskeletal injury. Twenty-three HIAs were excluded from the analysis owing to insufficient information to confirm the diagnosis from the HIA records and/or video footage after independent review. Twenty-four further HIAs were ineligible for inclusion, as the samples failed quality control checks.

A total of 1028 participants were recruited to the study (figure 1). In season 1 (2017–2018), players from 11 (out of 12) England’s Premiership (highest league) and 11 (out of 12) Championship (second league) clubs participated. One Championship club subsequently withdrew after the baseline sample collection owing to lack of resources to support the study. In season 2 (2018–2019) players from 11 (out of 12) Premiership clubs took part. Championship clubs were involved in a separate evaluation of concussion prevention.^31^ During and after games, following evaluation using the HIA protocol, participants for whom concussion was confirmed were categorised as HIA+ and participants for whom concussion was ruled out were categorised as HIA−. Uninjured players from the same game, matched for number of minutes played (uninjured controls), and players removed owing to musculoskeletal injuries (MSK controls) provided control samples. Participant characteristics are shown in table 1.

**Table 1:** Participants’ demographics

Characteristics	HIA+ group (n=106)	HIA− group (n=50)	Uninjured group (n=102)	MSK group (n=66)	Test statistic F or Χ^2^	P value
Age, mean (SD), years	27.1 (4.0)	27.5 (3.4)	26.4 (4.1)	25.9 (3.6)	2.25	0.08
Height, mean (SD), cm	186 (7)	188 (7)	186 (8)	187 (6)	0.43	0.73
Weight, mean (SD), kg	104.1 (11.9)	106.5 (12.7)	105.3 (12.7)	106.3 (12.2)	0.57	0.64
Minutes Played, mean (SD), min	37.8 (23.1)	54.6 (18.6)	52.8 (27.3)	55.6 (23.1)	10.38	*0.00
Ethnicity, No (%)						
White (British/Irish/other)	72 (82%)	35 (76%)	74 (84%)	48 (84%)		
Black or black British	2 (2%)	2 (4%)	3 (3%)	3 (5%)		
Other ethnic group	14 (16%)	9 (20%)	14 (15%)	6 (11%)		

Age, height, weight and ethnicity (where disclosed) of participants.

#### Study design

Saliva samples were collected preseason (baseline, B). During games, if a player required an HIA, saliva was collected per protocol. World Rugby (the International Federation) sanctioned an extension of the time permitted for an in-game HIA assessment from 10 min to 13 min to allow for saliva collection. In-game samples (T1), were followed by post-game (T2) and 36–48 hours post-game (T3) samples. Uninjured and MSK controls provided samples at T2 and T3 time points. In season 2, samples were not collected at T1 because sufficient data at this time point had been collected in season 1, and because T2 is the pre-established time point of the study.^30^

#### HIA protocol

The HIA protocol has been described in detail,^28^ and is a three time point multimodal assessment process performed by trained team physicians that incorporates: (i) HIA1: in-game, immediate removal of players showing clear signs of concussion, or, where a meaningful head impact has occurred without clear signs of concussion, a temporary substitution to allow the team physician to perform an off-field medical assessment aligned with the Sports Concussion Assessment Tool version 5 (SCAT5),^32^ (ii) HIA2: post-game medical assessment within 3 hours of a head impact supported by the SCAT5, and (iii) HIA3: medical assessment 36–48 hours after a head impact to monitor clinical progress and to confirm or refute the diagnosis of concussion, supported by the SCAT5. If there is no suspicion of concussion following the HIA1, the player can return to play, but they still complete HIA2 and HIA3 assessments. All medical staff involved in the delivery of the HIA protocol undertake annual mandatory training and weekly formal review (supported by video); governance and disciplinary processes are in place to monitor compliance. In order to ensure a consistent diagnostic standard for the study, at the end of each season the full HIA protocol documentation for each player assessed for concussion and (where available) the video footage of the inciting head injury were reviewed independently against the HIA protocol by two experienced sports medicine doctors. They were blinded to any laboratory results and adjudicated each incident as HIA+ or HIA− or recommended exclusion of the incident due to insufficient or conflicting evidence. This resulted in the exclusion of 47 HIAs (figure 1).

#### Saliva collection

Saliva (2 mL) was collected in by passive drool in Oragene-RNA RE-100 saliva self-collection kits (DNA Genotek) containing an RNA stabilising solution preserving the samples for up to 8 weeks. In the laboratory, samples were processed according to the manufacturer’s protocol for storage. During the second season, the RE-100 kits were discontinued and replaced with an equivalent product (CP-190), which was used from January 2018.

### Biomarkers identification

#### Next generation sequencing

In order to discover any sncRNAs that might be implicated in the response to concussion in an unbiased way, NGS was performed using 15 baseline samples, 15 HIA+ post-game (T2) samples and 20 control T2 samples (10 MSK and 10 uninjured controls) from season 1. The false discovery rate was minimised using the Benjamini-Hochberg procedure, which allowed the strongest candidates, in terms of fold change and significance, to be taken forward for quantitative PCR (qPCR) analysis. No fold-change cut-off point was selected. Details of the full NGS procedure are provided in the online supplemental material.

10.1136/bjsports-2020-103274.supp1 Supplementary data

#### sncRNA qPCR data analysis in season 1

To measure accurately the candidate biomarkers identified by the NGS, qPCR analysis was carried out on all available samples from season 1 at QIAGEN Genomic Services, (Germany). This included 393 in-match and post-match samples and 176 corresponding baseline samples. qPCR normalisation was performed based on the average of hsa-miR-29c-3p and hsa-let-7b-5p, the two most stable miRs identified across all samples by Normofinder software. The full qPCR procedure is described in the supplementary material.

Normalised ΔCq values were checked for normal distribution and then two-tailed independent-samples t-tests were performed to compare means of pair groups (HIA+ vs HIA− at T1, T2 and T3; HIA+ vs uninjured at T2 and T3; HIA+ vs MSK at T2 and T3; univariate analysis). In HIA+ samples, two-tailed paired-samples t-tests were performed to compare the means of baseline and concussion levels of the same subjects. Accuracy is reported as area under the curve (AUC) on a receiver operating characteristics plot.

### Time course of concussion biomarkers

Analysis of variance was performed in HIA+ and HIA− groups over time (baseline, T1, T2 and T3). Comparisons across multiple time points were evaluated using post-hoc Tukey’s honestly significant difference test. A corrected p<0.05 was considered significant.

### Biomarker validation and predictive model

#### sncRNA qPCR data analysis in season 2

In season 2, all significantly expressed biomarkers identified in season 1 across the different comparisons (HIA+ vs HIA−; HIA+ vs uninjured; HIA+ vs MSK) were analysed on a total 137 samples by qPCR as described in the supplementary material. t-Tests were performed for each group comparisons.

#### Development and testing of the multivariate predictive model

The concussion classification accuracy of the sncRNAs selected in season 1 (HIA+ vs HIA−, uninjured and MSK combined at T2) was evaluated using stepwise forward multivariable logistic regression analysis. This was used to select a panel of biomarkers from season 1 data (training dataset) to combine in a predictive algorithm using the logit function (intercept+β_1_Χ _1_+ β_2_Χ_2_…+ β_14_Χ_14_, where β is the unstandardised beta weight and Χ is the measured concentration for the biomarker), and calculate the predicted probability (e^∧^logit/(1+e^∧^logit)) and group membership (cut-off point ≥0.5 probability for concussion) for each player. The algorithm was subsequently prospectively tested on the independent season 2 data (test dataset) by replacing Χ_i_ with the new biomarker concentration values and blindly calculating predicted probability and group membership for each player. Goodness of fit was confirmed with the Hosmer-Lemeshow test. Accuracy was determined by measuring the AUC of the predicted group membership against the actual membership for the combined panel. Demographic data (age, height, weight) were then included in the regression model as covariates.

### Target prediction and KEGG pathway analysis

To evaluate the plausibility of the microRNA biomarkers identified by this analysis, their biological targets and Kyoto Encyclopedia of Genes and Genomes (KEGG) pathways were assessed using the mirPath v.3 tool on DIANA tools via microT-CDS (v5.0) prediction,^33^ and selecting the following criteria: false discovery rate correction; p value threshold <0.05; MicroT threshold 0.8.

## Results

### Data collection

Across the two seasons 393 HIAs were conducted in 184 players. Samples from 47 HIAs on 28 individual players were excluded as either ineligible or after independent review of the HIA records and video footage. Following clinical assessment, concussion was confirmed (HIA+) in 106 players and ruled out (HIA−) in 50. Compliance was 56% in both seasons.

### Biomarkers identification

#### Next generation sequencing and data reduction

In season 1, NGS identified 38 known microRNAs, 233 putative-miRs (put-miR) and 168 other small RNAs as being differentially expressed at T2 between HIA+ samples and MSK/uninjured control samples (online supplemental material eTable 1).

**Figure 2:**
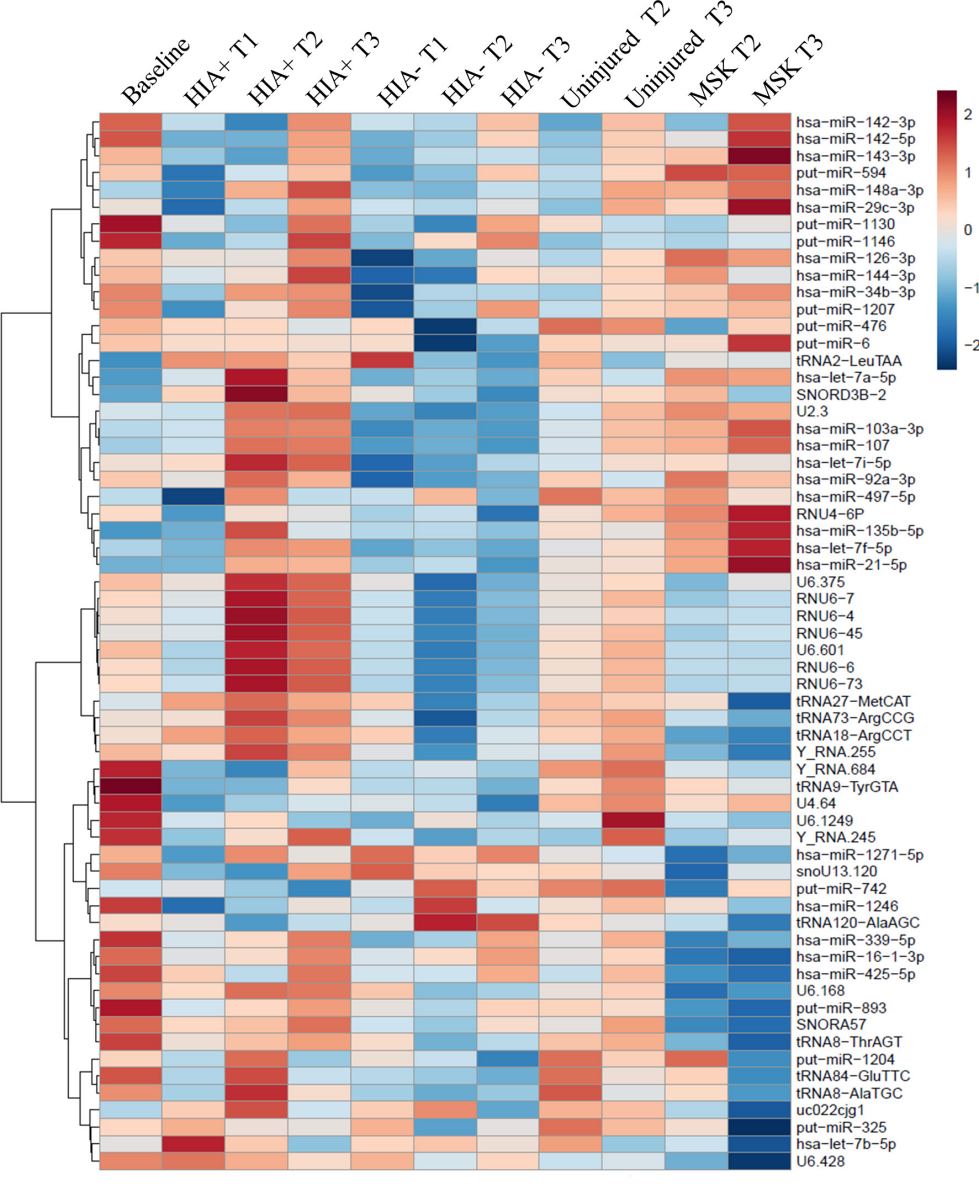
Heat map representing the average value of the concentrations of the miRNAs across different groups. Hierarchical clustering was performed across groups and miRNAs to check the similar behaviour of the miRNAs. HIA, head injury assessment; MSK, musculoskeletal.

HIA+ samples were then compared with HIA−, uninjured and MSK groups at different time points, as well as baseline samples, using qPCR. The initial analysis included 193 samples. Based on the strength of discrimination of concussed subjects, 32 known microRNAs, 34 put-miRs and 28 other small-RNAs were selected for further analysis in 376 further samples (ie, 569 samples in total). Of the 94 sncRNAs, 31 had >30% missing values and were removed. Among the remaining 63 sncRNAs, listed in online supplemental material eTable 2, the percentage of missing data was low (mean 3.2%, median 0%). These were used for statistical comparisons between the different groups and time points. A heat map representing the average value of the concentrations of the sncRNAs across different groups and sample time points is shown in figure 2.

#### sncRNA qPCR data analysis in season 1.

**Figure 3:**
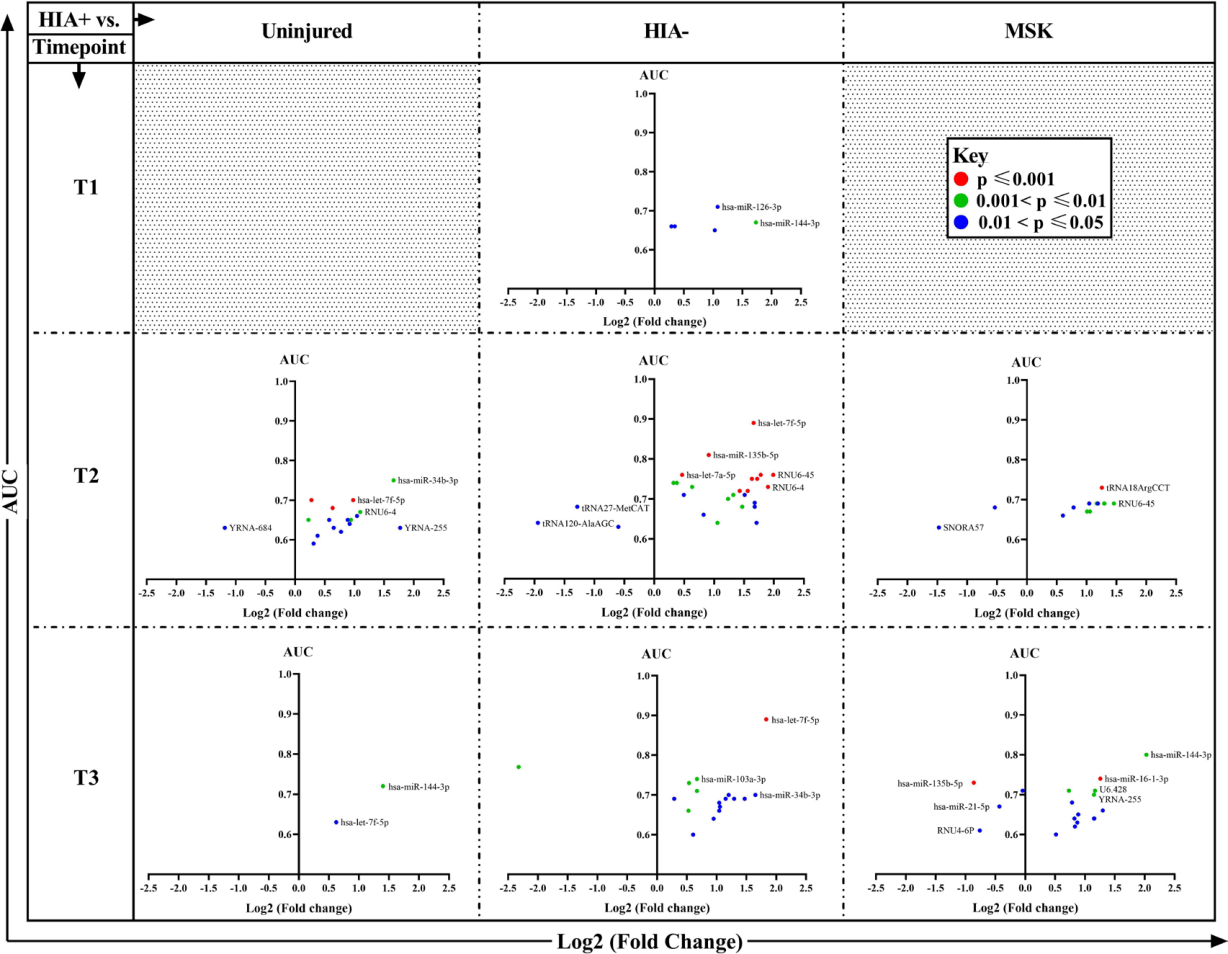
Plots of comparisons between players with a head injury (HIA+; n=106) and non-concussed groups (uninjured (n=102), HIA− (n=50) and musculoskeletal (n=66) groups)) at all time points. The y-axis represents the area under the curve (AUC) and the x-axis the log 2 expression of fold change of significantly differentially expressed sncRNAs between HIA+ and the other groups at each time point. The colour identifies the p values of the t-test analysis of each sncRNA (red ≤0.001, green ≤0.01 and >0.001, and blue ≤0.05 and >0.01). The full analysis results (AUC, CI, count, ΔCq average, SD, ΔΔcq, fold change and t-test p value) are available in the online supplemental materials. MSK, musculoskeletal; sncRNAs, small non-coding RNAs.

A large number of sncRNAs were significantly over- or underexpressed in concussed players compared with the other groups, at all post-injury time points, with several members of the let-7 and RNU6 families standing out in this analysis. The results are illustrated diagrammatically in figure 3. Let-7f-5p presented the highest AUC (0.89) and p value <0.001 at both time points T2 and T3.

The full data on AUC, CI, count, ΔCq (mean±SD), ΔΔcq, fold change, t-test p value and power analysis for each comparison of individual biomarkers at time point T1, T2 and T3 are provided in the supplementary material (online supplemental material eTable 3–6).

### Biomarker time course

**Figure 4:**
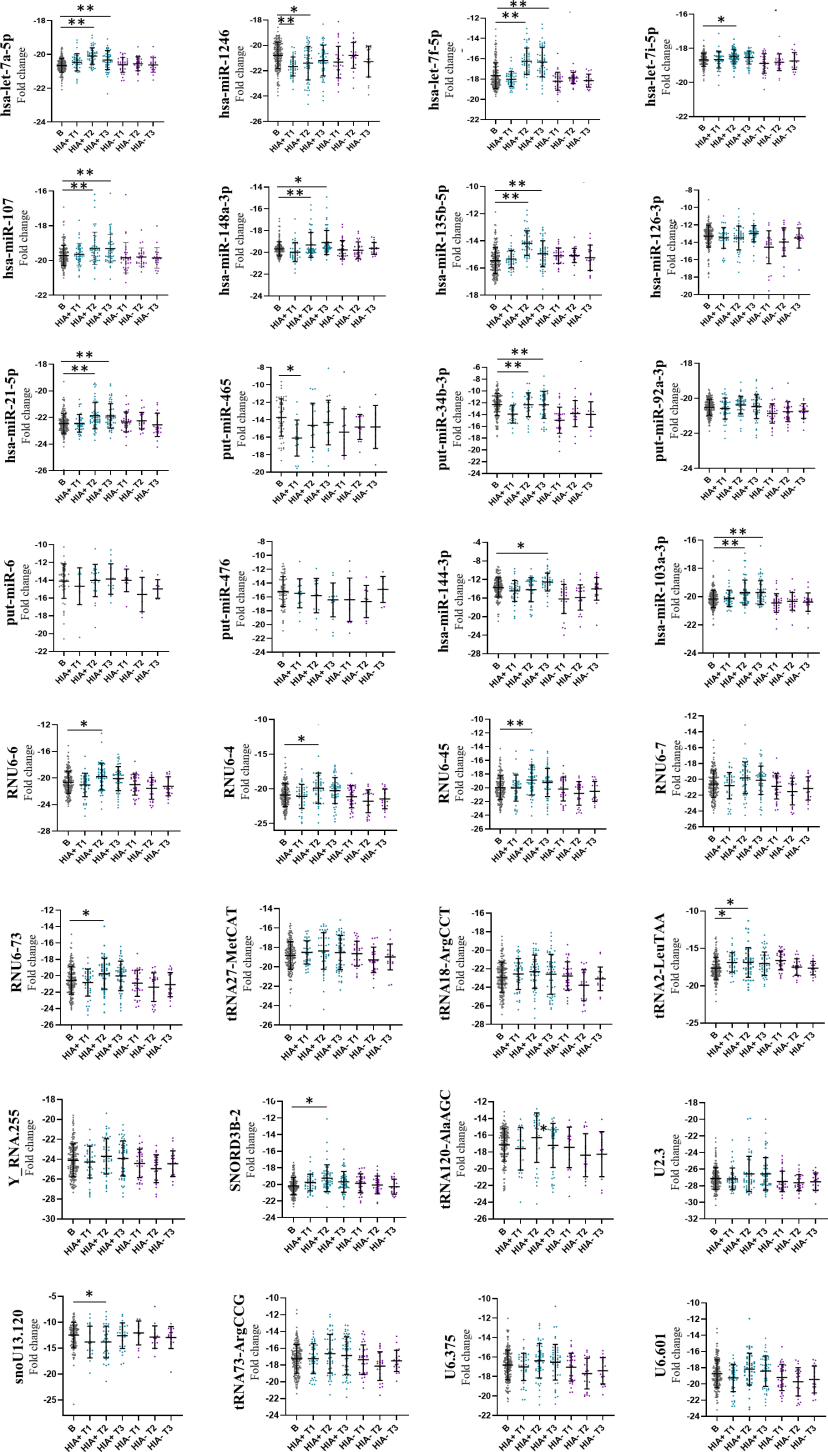
Longitudinal analysis. Thrty-two biomarkers selected as differentially expressed in the comparison HIA+ versus HIA− were used for the longitudinal analysis. Analysis of variance was performed in HIA+ and HIA− groups over time (T1, T2 and T3) and compared with baseline. Comparisons across multiple time points were evaluated using post-hoc Tukey’s honestly significant difference test. *Significantly different from baseline p<0.05; **significantly different form baseline p<0.001. HIA, head injury assessment.

Thirty-two different biomarkers were identified as differentially expressed between HIA+ and HIA− groups when compared at T1, T2 and T3 (online supplemental eTable 3–5). These 32 biomarkers were longitudinally evaluated across the different time points with reference to their baseline values. In the concussed group, as shown in figure 4, there was significant interaction of several biomarkers with time from injury, with 14 sncRNAs overexpressed and five underexpressed following concussion with respect to baseline values. The greatest effect of time (p<0.001) was found for let-7a-5p, let-7f-5p, miR-107, miR-148a-3p, miR-135b-5p, miR-21-5p, miR-34b-3p, miR-103a-3p and RNU6-45, which were overexpressed, and miR-1246, which was underexpressed. Conversely, in the HIA− group the biomarkers remained much closer to their baseline values throughout all time points after injury.

### Biomarker validation and predictive model

#### sncRNA qPCR data analysis in season 2

To validate the candidate sncRNAs prospectively, 137 saliva samples were analysed by qPCR in season 2. Although the sample size of season 2 was smaller, several biomarkers found to be differentially expressed between groups in season 1 were confirmed to be statistically significantly different across the same comparisons. Results are reported in online supplemental eTables 3–5. Let-7f-5p was confirmed as discriminating between HIA+ and HIA− (p=0.007) with an AUC of 0.80 (95% CI 0.62 to 0.97) at 36–48 hours only.

#### Multivariate predictive model performance

**Figure 5:**
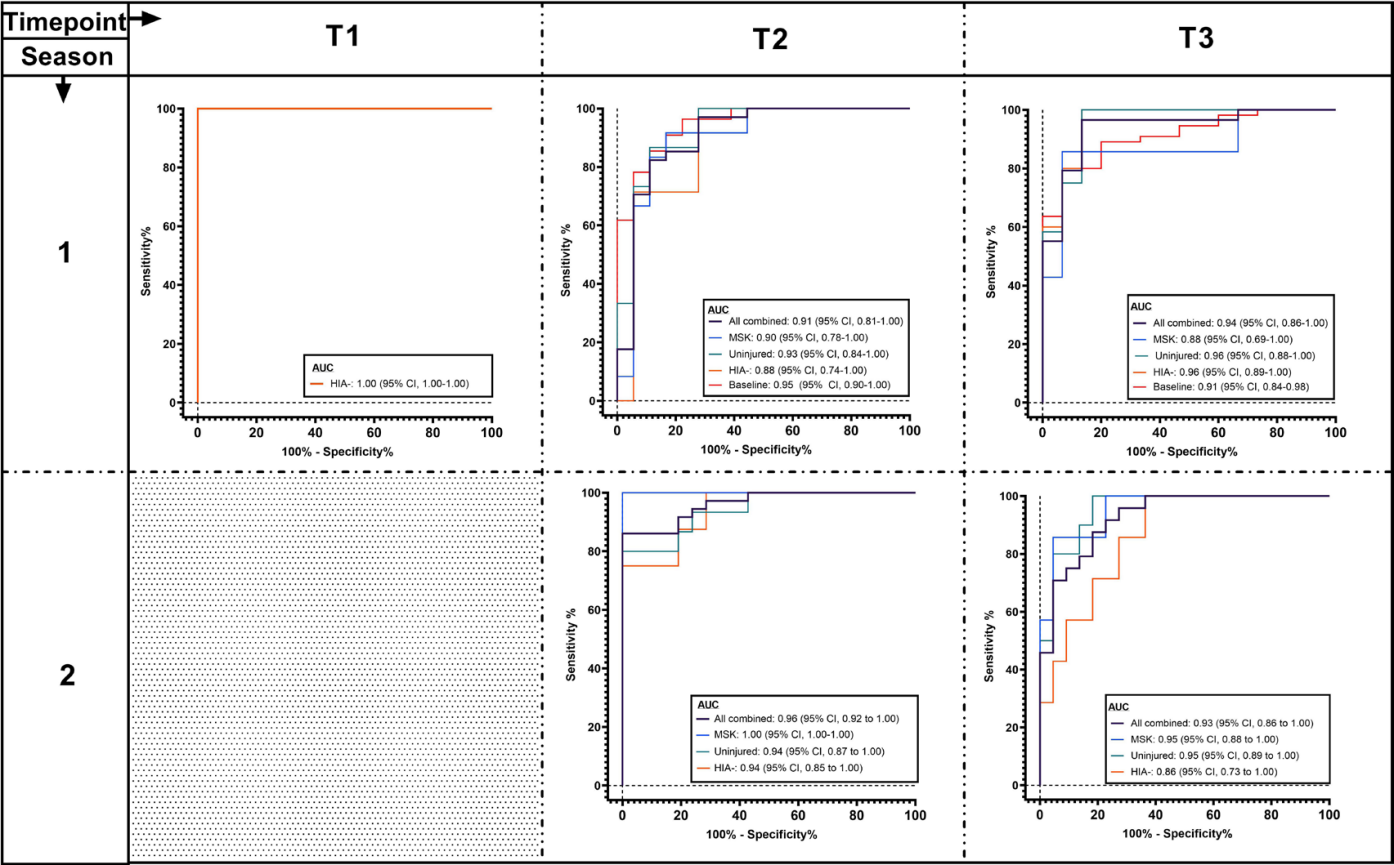
Receiver operating characteristic curve results for a panel of 14 combined small non-coding RNAs differentiating players with confirmed concussion after the head injury assessment (HIA+) from other groups, including players who underwent a head injury assessment but had concussion ruled out (HIA−, orange line); Uninjured players from the same game who played a comparable number of minutes to those of the HIA players (uninjured, green line); players who were removed from the game due to musculoskeletal injuries (MSK, blue line); HIA−, uninjured and MSK groups combined (all combined, black line); and preseason values for concussed players (baseline, red line). The curves are shown for all time points (T1=during the game; T2=immediately after the game), and T3=36–48 hours after the game). Season 1 represents the training dataset and season 2 the test dataset (HIA+, HIA−, uninjured and MSK groups, as well as HIA−, uninjured and MSK groups combined) for the logistic regression model. Samples were not collected during the game in season 2.

The logistic regression analysis identified 14 biomarkers (let-7a-5p, miR-143-3p, miR-103a-3p, miR-34b-3p, RNU6-7, RNU6-45, Snora57, snoU13.120, tRNA18Arg-CCT, U6-168, U6-428, U6-1249, Uco22cjg1 and YRNA_255) as offering the highest accuracy. The algorithm of the combined 14-biomarker panel showed accuracy of 0.91 (95% CI 0.81 to 1, p<0.001) at differentiating concussion (HIA+) from other groups (HIA−, uninjured and MSK combined) at T2 (figure 5 and online supplemental eTable 7) in the training dataset.

When prospective tested on season 2 (test dataset), the algorithm was highly accurate in classifying HIA+ from controls (HIA−, uninjured and MSK combined) at T2 (AUC 0.96, 95% CI 0.92 to 1, p<0001) and T3 (AUC 0.93, 95% CI 0.86 to 1, p<0.001). Summary statistics for the 14 biomarkers are presented in online supplemental eTable 8. The inclusion of demographic data in the model showed no evidence of multicollinearity.

### Target prediction and pathway analysis

KEGG pathway analysis was performed using the microRNAs identified in the comparison HIA+ vs HIA− at T1, T2 and T3. The pathway analysis revealed a strong association between let-7 family members and the extracellular matrix (ECM) receptor interactions pathway, as well as a strong relation between miR-103a-5p and miR-107 and fatty acid metabolism/biosynthesis. The full results are presented in online supplemental eFigures 1, 2 and 3.

## Discussion

Current evaluation of sport-related concussion is largely based on the reporting of symptoms. Biomarkers are not used in this field at present but may prove useful for corroborating the diagnosis. In this study, we sought to investigate the role of salivary sncRNAs as a new class of molecules to serve as potential biomarkers of sport-related concussion. We assessed these against a standard tool used to diagnose concussion in professional rugby. It is important to note, however, that biomarkers are the expression of biological changes, which may correlate with clinical outcomes, just as clinical outcomes may be reasonable proxies for underlying biological changes, but that these are not replacements for each other.

We identified 32 different sncRNAs across three time points that were differentially expressed in rugby players with a clinical diagnosis of concussion compared with those cleared of concussion after examination. In particular let-7f-5p offered good discrimination between HIA+ and HIA− (AUC 0.89 in season 1 and AUC 0.80 in season 2) at 36–48 hours. We also identified significant dynamic changes in the concentration of sncRNAs between time points post-concussion, suggesting that the biological response to injury evolves rapidly, as far as sncRNAs and the cellular processes that these post-transcriptionally regulate are concerned.

Finally, the key finding of this study was the identification of a panel of 14 different biomarkers that accurately predicted clinical diagnosis of concussion in professional rugby players (with an AUC of 0.96 for the classification of HIA+ from controls immediately post-game), when prospectively tested on independent data.

Taken together, sncRNAs have the potential to provide insight into pathophysiological responses in concussion and to be potentially clinically useful in the diagnosis of concussion.

The biological plausibility of the biomarkers is corroborated by the notion that among the identified biomarkers, several have previously been associated with concussion/TBI or related pathologies, such as mir-21, an anti-inflammatory regulator, playing crucial role in the central nervous system^34–36^; miR-144, which promotes β-amyloid accumulation by suppressing ADAM10 expression^37^; miR-1246 recently described as potential candidate of concussion^21^; miR-135b-5p previously identified by our group as salivary biomarker of concussion^19^ and the let-7 family, including let-7i-5p, let-7b, let-7a-3p, let-7c-5p,^19 22 23 38 39^ which is an important family of neuroinflammatory modulators showing an involvement in Alzheimer’s disease and in major depressive episodes.^40 41^ Bioinformatically, we showed a strong association between let-7 family members and the ECM receptor interactions pathway. Changes in levels of members of the let-7 family were reported in previous studies in concussion, and integrity and changes in the ECM have been observed in brain injury,^42^ and other neuropathologies.^43^ Another strong relation between miR-103a-5p and miR-107 and fatty acid metabolism/biosynthesis was also evident. This is intriguing, as a shift from glucose to fatty acid metabolism has been described in the transcriptomic response to mild TBI.^44^ These initial findings highlight the potential for sncRNAs in developing a greater understanding of the pathophysiological response to concussion.

SncRNAs are stable and straightforward to assess. In addition, the choice of saliva as a non-invasive fluid allows for rapid and well-tolerated pitch-side and post-game collection at very early and specific time points. It is proposed that saliva can receive exosomal miRNAs directly from cranial nerves in the oropharynx, and as such there is a rapid response within saliva after TBI, making them particularly suitable for a pitch-side diagnosis.^45^ The development of point-of-care testing for salivary sncRNAs would offer the prospect of a test for concussion that can support clinical decision-making in sport.

### Strengths and limitations

It is important to note that although the number of players recruited to participate in the study is over 1000, the number of players who are regular starting players in each team, exposed to potential concussions every week, is much smaller. Therefore, a small number of players appeared more than once in different groups or less frequently in the same group. This can positively or negatively affect the magnitude of group differences.

Including uninjured players and players with musculoskeletal injuries as controls avoids the pitfalls of some previous biomarker research by ruling out the possibility that putative concussion biomarkers may come from other sources. We also analysed the baseline preseason samples of cases and controls to account for the potential influence of pre-existing factors. This led to the selection of a narrow panel of sncRNA biomarkers that are likely to represent a true signature of concussion. As sncRNAs appear to act in a concerted manner, analysis of multiple interacting pathways is likely to provide a more accurate description of the response to a specific insult than individual biomarkers. This adds complexity when constructing a test, with an inherent risk of overfitting when testing multiple possible combinations. We sought to reduce the risk of overfitting by confirming the expression of our biomarkers across independent datasets from two separate seasons.

The study benefitted from not using a selected sample but instead recruiting all available players who were exposed to head injury with the potential to result in concussion from 22 of 24 of the clubs participating in the top two tiers of English professional rugby. On the basis that participation was voluntary, and given the complexity of carrying out research procedures in a competitive sport environment, we were pleased with the overall 56% compliance. However, we cannot ascertain whether the remaining incidents were missed at random or systematically and how the results might have changed if samples had been included from these. For the clinical translation of the study, we would of course expect compliance to be much higher if these procedures were formally a part of a concussion management plan, as opposed to a research protocol.

The HIA protocol, used here as the current operational ‘gold standard’ for the diagnosis of concussion in elite rugby union, provides the clinical outcome comparison for the assessment of the biomarkers. The standardised clinical assessment, diagnosis and reporting of concussion by team physicians specifically trained in the application of the HIA protocol, with subsequent independent review of all cases, provides a high standard of clinical evaluation of concussion, plausibly explaining the good concordance between clinical diagnosis and biomarker expression. It is clear that in this study design the biomarkers cannot outperform the clinical assessment. As a consequence, we believe the biomarkers are likely to prove most useful in non-professional sport settings, where the overwhelming majority of sport-related concussions occur. In these settings, access to trained healthcare professionals is limited both during and after the game and it is recommended that the presence of a single symptom or sign of concussion in the context of a head injury should result in concussion being suspected.

Finally, a limitation of the study is that it includes only male elite players, which could potentially affect the generalisability of its findings.

## Conclusion: implications for the field

The detection of signatures of concussion at early time points in saliva (a non-invasively sampled biofluid) presents both at the pitch side, and in primary care and emergency medicine departments, an opportunity to develop a new and objective diagnostic tool for this common clinical presentation. In addition, sncRNAs may be an important tool in developing understanding of the pathophysiology of concussion.

**Summary box**

**What are the findings?**

- This study, conducted in a professional contact sport setting, has identified and prospectively validated both single candidates and a panel of small non-coding RNA salivary biomarkers of concussion.
- Let-7f-5p could distinguish players with confirmed concussion from those for whom concussion was subsequently ruled out after structured head injury assessments with an area under the curve (AUC) up to 0.89 at 36–48 hours from injury.
- A combination of 14 salivary biomarkers was highly accurate (AUCs 0.96 immediately post-game and 0.93 at 36–48 hours post-game) at identifying concussed players from all other groups, including players with suspicion of traumatic brain injury who had a concussion ruled out after a structured head injury assessment, uninjured controls from the same game and players who had had musculoskeletal injuries.

**How might it impact on clinical practice in the near future?**

- The biology of concussion is still not fully understood. Small non-coding RNAs provide further insights into the response to injury as this evolves from immediately after the event to several hours later.
- Concussion can be hard to diagnose and is often missed, especially where a structured evaluation by an expert clinician is not possible—for example, at grass-root level. Small non-coding RNAs can provide a diagnostic tool that might reduce the risk of missing this type of injury at all levels of participation.
- In community sport, salivary small non-coding RNAs may provide a non-invasive diagnostic test that is comparable in accuracy to the level of assessment available in a professional sport setting.
- At an elite level of participation, this diagnostic tool may become an adjunct to current head injury evaluation protocols.

## Data Availability

Data are available upon reasonable request. All data relevant to the study are included in the article or uploaded as supplementary information. Deidentified participant data available on request. Please contact the corresponding authors at the following address: v.dipietro@bham.ac.uk; a.belli@bham.ac.uk.
