# Supplementary material for: Unique diagnostic signatures of concussion in the saliva of male athletes: the Study of Concussion in Rugby Union through MicroRNAs (SCRUM)
Source: Br J Sports Med. 2021 Mar 23;55(24):1395–404. doi: 10.1136/bjsports-2020-103274 (PMC8639909; doi:10.1136/bjsports-2020-103274)
Supplement: Supplementary data [file bjsports-2020-103274supp001.pdf]

Supplementary Materials

Procedures

Two ml of saliva was passively collected from all subjects at clubs competing in the top two tiers of male Rugby Football Union in England across the 2017-18 (Premiership and Championship clubs) and 2018-19 (Premiership clubs only) seasons. Samples collected pre-season were used as baseline. During the season, samples were collected when club medical staff assessed players for concussion following head injury events with the potential to result in concussion using the standardised World Rugby Head Injury Assessment (HIA) protocol. [1] The key features of the protocol, that is in widespread use in elite professional rugby, are mandatory standardised assessments, aligned with the Sports Concussion Assessment Tool (SCAT5) at three time points informing a team physician's clinical diagnosis. A definitive diagnosis of concussion is made if a player demonstrates observable signs of concussion at the time of the head injury event, requiring immediate and permanent removal from play (e.g. loss of consciousness, tonic posturing or ataxia) or a clinical diagnosis of concussion is made supported by the standardised (SCAT5) assessment performed post game and/or 36-48 hrs later. The interpretation of the SCAT5 assessment is with reference to individual baseline values and any negative deviation from baseline performance is recommended as strongly suggestive of a diagnosis of concussion, though the clinical judgment of the team physician takes precedence over the performance in the SCAT5 assessment. If the defined observable signs of concussion are nor clearly seen following a witnessed head injury event however, and the diagnosis of concussion is uncertain, a player may be temporarily substituted to allow for a standardised in match but off field assessment to be conducted by the team physician. If concussion is then excluded by the team physician, there is an ability for the player to return to

play. World rugby rules required this assessment to be completed within 10 minutes, but a special dispensation was given for an extension of 3 minutes to permit study procedures to be carried out as part of the protocol for the current study in season 1.

Annual mandatory training programmes must be undertaken by all medical staff involved in the delivery of the HIA protocol. Formal audit, governance and disciplinary processes are in place to monitor compliance. All assessments are entered by team medical staff in real-time on an app (CSx) (https://csx.co.nz/our-story/) and are available for subsequent audit and review.

The HIA protocol incorporates a very clearly defined and replicable definition of what constitutes a sport-related concussion and provided the diagnostic reference for our analysis. For the purpose of this study, we defined the in-match assessment as time point T1, the post-match assessment as time point T2 and the 36-48 hour assessment as time point T3.

Participants who were evaluated using the HIA protocol formed the HIA+ group if concussion was confirmed at any of the three time points, and the HIA- group if concussion was initially considered but subsequently ruled out.

Whenever a participant was assessed post-match (T2) for possible concussion, team medical staff were asked to identify another participant who had played a similar number of minutes in the same match but who had not had been assessed for concussion and, if possible, a third participant who had had to be withdrawn from that match due to a musculoskeletal injury. Samples were requested from all categories of player at near enough the same time after the final whistle and were completed before players finished getting showered and changed.

These provided samples at time points T2 and T3, to form the uninjured and the musculoskeletal Injury (MSK) control groups respectively.

The HIA-T2 assessment was usually carried out between 30-90 minutes after the match finished for the player. Therefore, the range of HIA-T2 assessments was from 30 minutes

through to 190 minutes (90 minutes+80 minutes playing time+20 minutes interval) post injury if a player was removed in the first or completed last minute of the game respectively.

It is also important to note that not all players had an assessment at time point T1, as not all significant head injury events are identified in game and symptoms for some players only develop post-game. Moreover, it would have not been possible to obtain samples from the uninjured group during the match, therefore, it was predetermined in the study design that T2 would be the primary time point of interest for comparisons, as this would provide the most consistent timeframe to collect saliva samples across all groups.

Although team physicians were responsible for the clinical management of each player in real-time, in order to ensure a consistent diagnostic standard for the study, the full HIA protocol documentation for each player assessed for concussion and (where available) the video footage of the inciting head injury event were subsequently reviewed independently against the HIA protocol by two experienced sports medicine doctors and England Senior National Team doctors (SPTK and RT). They were blinded to any laboratory results and adjudicated each incident as HIA+ or HIA- or recommended its exclusion due to insufficient or conflicting evidence. For completeness, the analysis of the uncensored data is presented in this section.

Saliva collection

Medical staff at the respective clubs were trained in the collection procedure. Saliva was collected in Oragene®-RNA RE-100 saliva self-collection kits (DNA Genotek) containing an RNA stabilizing solution preserving the samples for up to 8 weeks. Saliva was collected from each participant at enrolment and at the time points described above. Samples were transported to the lab in Birmingham, where they were processed in line with the manufacturer's protocol for storage. During the second season, DNA Genotek discontinued the RE-100 kits and replaced them with an equivalent product (CP-190). This was utilised from January 2018 onwards.

NGS procedure

Library preparation and Next Generation Sequencing

Library preparation was carried out using the QIAseq miRNA Library Kit (QIAGEN). A total of 5ul total RNA was converted into microRNA NGS libraries. Adapters containing UMIs were ligated to the RNA. Then RNA was converted to cDNA. The cDNA as amplified using PCR (22 cycles) and during the PCR, indices were added. After PCR the samples were purified. Library preparation QC was performed using either the Bioanalyzer 2100 (Agilent) or TapeStation4200 (Agilent). Based on quality of the inserts and the concentration measurements the libraries were pooled in equimolar ratios. The library pool(s) were quantified using the qPCR ExiSEQ LNA™ Quant kit (Exiqon). The library pools were then sequenced on a NextSeq500 sequencing instrument according to the manufacturers instructions (NEBNext Multiplex Small RNA Library Prep Set for Illumina) to make approximately 163-175 base-pair sized libraries. Raw data as demultiplexed and FASTQ files for each sample were generated using the bcl2fastq software (Illumina inc.). FASTQ data were checked using the FastQC tool (http://. bioinformatics.babraham.ac.uk/proeects/fastqc/).

Mapping

A reference profile of sequencing data for each sample was obtained using the whole human genome sequence GRCh37, downloaded from the Genome Reference Consortium and mirbase_20 as an annotation reference. Reads were aligned to the miRbase using Bowtie2.[2] The mapping criteria for aligning reads to spike-ins, abundant sequence and miRBase were the reads to have perfect match to the reference sequences. For mapping to the genome, the restricting was one mismatch which was allowed in the first 32 bases of the read. No in-dels were allowed in mapping. Unaligned reads were mapped against the host reference genome and used as input for mirPara [3] and miRbase to predict putative miRNAs. [4,5]

Statistical analysis

P-values for significantly differentially expressed sncRNAs are estimated by an exact test on the negative binomial distribution. Aligned reads were counted and differential expression analysis, p-values for significantly differentially expressed microRNAs and false discovery rate according to Benjamini-Hochberg were performed with EdgeR.[6] For normalisation, the trimmed mean of M-values (TMM) method based on log-fold and absolute gene-wise changes in expression levels between samples was used.

qPCR season1

14 µl RNA was reverse transcribed in 70 µl reactions using the miRCURY LNA RT Kit (QIAGEN). cDNA was diluted 50 x and assayed in 10 µl PCR reactions according to the protocol for miRCURY LNA miRNA PCR; each miRNA was assayed once by qPCR on the miRNA Ready-to-Use PCR, custom panel using miRCURY LNA SYBR Green master mix. qPCR Probes are the complementary sequences of the sncRNAsof interest (eTable 2 below).

Negative controls excluding template from the reverse transcription reaction was performed and profiled like the samples. The amplification was performed in a LightCyclerp 480 Real-Time PCR System (Roche) in 384 well plates. The amplification curves were analysed using the Roche LC software, both for determination of Cq (by the 2nd derivative method) and for melt curve analysis. The amplification efficiency was calculated using algorithms similar to the LinReg software. All assays were inspected for distinct melting curves (Tm) and the Tm was checked to be within known specifications for the assay. Furthermore, assays must be detected with 0 Cq less than the negative control, and with Cq<37 to be included in the data analysis. Data that did not pass these criteria were omitted from any further analysis. Cq was calculated as the 2nd derivative. Normalization was performed based on the average of hsa-

miR-29c-3p and hsa-let-7b-5p (custom normalizer assays), the two most stable miRs identified across all samples by Normofinder software.[7]

The formula used to calculate the normalized Cq values is the difference between the custom normalizer assays mean Cq and the assay Cq (miRNA of interest). After normalization 20 has been added to the normalized dCq values to shift the numbers in a positive range to allow using the qPCR analysis pipelins according Qiagen procedures. While processing the data in the qPCR pipeline a minus is inserted before the normalized dCq value. A higher value indicates that the miRNA is more abundant in that sample.

qPCR season 2

RNA from saliva samples was extracted and analysed with exactly the same protocol used for SCRUM1 and qPCR was performed using the Applied Biosystems Quantstudio 5 (ThermoFisher Scientific) for amplification and melt curve analysis.

ADDITIONAL ANALYSIS RESULTS

NGS and qPCR data analysis

The validation was performed in 2 different qPCR steps. Initially, all fragments showing a different expression between HIA+ T2 and MSK+uninjured T2 groups in NGS analysis (eTable1), were selected for the first qPCR validation step. This list, comprising 38 known microRNAs, 233 put-miRs and 168 other small RNAs, was analysed in 193 samples. Following this initial analysis, based on the strength of discrimination of concussed subjects, we selected 32 known microRNAs (panel A); 28 other small RNAs (panel B) and 34 put-miRs (panel C) for further analysis in 376 independent samples The Cq values obtained from the 2 qPCR validation sets were finally merged and collected in a total of 569 samples. Of the 94 sncRNAs,

31 had >30% missing values and were removed. The remaining 63 sncRNAs (comprisinf og 23 microRNAs, 11 putative microRNAs and 29 other small non-coding RNAs) are listed in eTable2 and were used for statistical comparisons between the different groups and time points (eTable 3,4,5,6).

The power analyses indicating how many samples are needed per group in a follow-up study to validate the results from the present analysis was calculated at a 0.95 level of confidence and for target p-value 0.05.

eTable 1: List of microRNAs (Panel A), other small non-coding (Panel B) and putative* microRNAs selected from NGS analysis.

*putative microRNAs are not previously described sequences and bioinformatically predicted as new microRNAs

Panel A

microRNAs	logFC	logCPM	p-Value	FDR
hsa-miR-199a-5p	-3.79380184	5.744164606	8.15823E-06	0.004797041
hsa-miR-133a-3p	-4.95698696	10.40020121	7.04586E-05	0.020714819
hsa-miR-206	-4.6959152	9.788488822	0.000180043	0.03528849
hsa-miR-1273h-3p	2.629420889	3.481729832	0.001297451	0.190725263
hsa-miR-133b	-4.20181151	6.465178373	0.002184888	0.256942851
hsa-miR-561-5p	1.579551555	4.525850808	0.003970504	0.348018465
hsa-miR-126-3p	-1.41838668	8.449681001	0.004143077	0.348018465
hsa-miR-16-1-3p	1.43849327	4.458454478	0.005764959	0.352449414
hsa-miR-449b-3p	3.467958972	3.218975	0.005920497	0.352449414
hsa-miR-449c-5p	2.749952622	5.534179256	0.006033974	0.352449414
hsa-miR-6748-3p	4.143101791	3.188086662	0.007073212	0.352449414
hsa-miR-133a-5p	-3.92797466	4.752927061	0.008472679	0.352449414
hsa-miR-34b-3p	2.1437498	4.339967792	0.008637715	0.352449414
hsa-miR-6824-3p	2.731145621	3.076936275	0.008970282	0.352449414
hsa-miR-5195-5p	5.376105018	4.09767258	0.009313959	0.352449414
hsa-miR-5096	2.906908779	4.723794715	0.00959046	0.352449414
hsa-miR-4488	3.491520052	3.097320079	0.012515423	0.409279535
hsa-miR-92a-3p	-0.60902412	10.49283489	0.012885103	0.409279535
hsa-miR-1	-2.36876254	10.80951287	0.013692259	0.409279535
hsa-miR-885-5p	1.507019382	4.182851751	0.013921073	0.409279535
hsa-miR-548h-5p	2.055544755	3.480848725	0.015148132	0.42414769
hsa-miR-1246	0.980484408	14.78271469	0.017085283	0.445746733
hsa-miR-4492	-3.18487947	3.53612341	0.017435672	0.445746733
hsa-miR-484	1.274418725	6.148369199	0.019071294	0.449461809
hsa-miR-449a	2.203777576	6.338766334	0.019109771	0.449461809
hsa-miR-3122	3.142476669	3.078975177	0.02000366	0.452390465

hsa-miR-1180-3p	1.165471248	3.889331639	0.021316281	0.457201951
hsa-miR-619-5p	2.792045377	3.207604869	0.021771521	0.457201951
hsa-miR-2277-5p	1.067026052	4.057818901	0.023836409	0.474930011
hsa-miR-5699-3p	1.876788488	2.955683933	0.024231123	0.474930011
hsa-miR-125b-2-3p	-0.96587973	4.933520984	0.025679562	0.487083301
hsa-miR-4449	2.06863176	3.047374886	0.027679506	0.503997815
hsa-miR-671-3p	1.125735449	3.934232773	0.02867908	0.503997815
hsa-miR-33a-3p	0.959071217	4.091785492	0.029142731	0.503997815
hsa-miR-193b-5p	-1.14025756	4.138245693	0.031271072	0.522692794
hsa-miR-34c-3p	1.690242737	4.489423918	0.0320016	0.522692794
hsa-miR-339-5p	1.057419584	7.902671962	0.034354837	0.545963351
hsa-miR-6813-3p	2.813650185	2.933287004	0.035310016	0.546376034
hsa-miR-671-5p	0.838663749	5.600518309	0.047339286	0.598988904
hsa-miR-1537-5p	1.129462606	4.117629615	0.048138455	0.598988904
hsa-miR-193a-3p	-1.5845588	3.995294293	0.048897053	0.598988904
hsa-miR-130a-3p	-0.88824466	6.580760266	0.051263632	0.604409433
hsa-miR-126-5p	-1.22112275	8.006357503	0.051621005	0.604409433

Panel B

small non-coding RNAs	logFC	logCPM	p-Value	FDR
tRNA138-ArgACG	-2.27538237	7.674009775	0.000243882	0.204980883
RNU6-11	-1.67718785	4.732492163	0.000245185	0.204980883
tRNA11-ArgACG	-2.21044709	7.59261144	0.000408122	0.204980883
RNU6-36	-1.84798113	4.63862015	0.000436362	0.204980883
tRNA175-SerGCT	-1.84762954	8.390549302	0.001054009	0.290869197
U3.39	4.655316415	4.424357984	0.001147835	0.290869197
RNU4-6P	-1.71160894	4.864935322	0.001317818	0.290869197
U6.375	-1.86391998	4.231358228	0.001366655	0.290869197
snoU13.63	4.286830869	4.29762358	0.001552935	0.290869197
tRNA8-ThrAGT	-1.65324248	9.428427256	0.001556007	0.290869197
RNU6-1	-1.37118776	4.843026936	0.001713194	0.290869197
RNU6-31	-1.53604238	4.741724921	0.002351551	0.290869197
tRNA4-ArgTCG	-1.70070174	10.2139807	0.002452994	0.290869197
tRNA130-ValCAC	-2.21562973	4.853113645	0.002797565	0.290869197
U3.3	-1.72401526	5.267608648	0.002991025	0.290869197
U4.57	-1.55456476	4.807782758	0.003084965	0.290869197
tRNA84-GluTTC	-1.39818637	10.32369425	0.003223874	0.290869197
U6.428	-1.83108856	4.169870903	0.003289229	0.290869197
U3.4	-1.80036039	5.421618508	0.00346512	0.290869197
tRNA95-AsnGTT	-2.24304788	4.752212371	0.003469246	0.290869197
uc003oif.3	-2.59243747	4.028384043	0.003838286	0.290869197
U3.2	-1.73251154	5.414684824	0.003880282	0.290869197
U3.42	2.126244843	4.003126274	0.003910676	0.290869197
uc002tgp.1	2.810368883	4.510093789	0.004221387	0.290869197
tRNA105-PseudoTTC	-1.59015632	7.983774255	0.004475105	0.290869197
tRNA4-GluCTC	-1.60952388	8.107675756	0.00453217	0.290869197
RNU6-14	-1.86375965	4.058373711	0.004721183	0.290869197
tRNA4-ThrAGT	-1.34446936	8.68050744	0.004730298	0.290869197
U6.97	-2.76317407	3.745083257	0.004815026	0.290869197
tRNA120-AlaAGC	-1.31815493	9.587766586	0.004875694	0.290869197
snoU13.348	2.256666202	4.075754187	0.005036202	0.290869197
U1.105	-2.32216301	4.207692971	0.005058512	0.290869197
tRNA27-MetCAT	-2.34782418	6.706470837	0.0051084	0.290869197
U1.10	-2.59329176	4.816351388	0.005540106	0.306172302
tRNA134-GluTTC	-1.3431528	10.39664851	0.006091168	0.32697053
RNU6-6	-1.54430018	4.19348578	0.00626447	0.32697053
Y_RNA.75	1.904869354	5.50161645	0.006713926	0.340251318
tRNA8-Undet	1.964251986	4.197405842	0.006905386	0.340251318
SNORA25	-2.35025006	3.740784336	0.007062161	0.340251318
RNU6-39	-1.60601357	4.333253034	0.007621329	0.3538182
tRNA119-AlaCGC	-1.25397272	9.514833568	0.007808074	0.3538182
tRNA85-PseudoTTC	1.126764653	5.8624175	0.008322426	0.3538182
U6.254	-1.80163898	3.954386151	0.008365638	0.3538182
tRNA3-ArgCCT	-1.40848109	10.46544955	0.008483646	0.3538182
SNORD114-2	-3.1187271	3.929437693	0.008576479	0.3538182
tRNA162-MetCAT	-2.31337858	6.829504657	0.008700411	0.3538182
Y_RNA.661	-2.13613309	3.924521406	0.008896949	0.3538182

tRNA8-AlaTGC	-1.03417608	10.23229845	0.009262877	0.3538182
tRNA56-ThrTGT	-1.43137722	10.38511882	0.009580922	0.3538182
tRNA26-AsnGTT	-1.2260783	8.800207822	0.009587969	0.3538182
tRNA73-ArgCCG	-0.87676773	7.646480047	0.009603368	0.3538182
tRNA156-ArgACG	-1.38659506	6.417990561	0.009974671	0.36043089
tRNA28-IleAAT	-0.96475102	8.290575814	0.010463073	0.370945534
RNU6-33	-1.41177741	4.713635974	0.010694162	0.372117242
tRNA3-ProTGG	2.564031745	13.63247318	0.01106572	0.37247176
tRNA19-ArgTCG	-1.57386692	9.439040466	0.011100808	0.37247176
tRNA164-MetCAT	-2.24158666	6.824859219	0.011731788	0.372701442
tRNA13-AlaCGC	-1.05980195	9.619304583	0.011862589	0.372701442
U6atac.4	-2.29659983	4.169967341	0.012205483	0.372701442
tRNA9-AlaAGC	-1.76859219	4.913678407	0.012206837	0.372701442
tRNA132-PseudoCAC	-2.28282945	4.122549605	0.012249051	0.372701442
RNU6-7	-1.54429239	4.25135459	0.012297759	0.372701442
tRNA20-MetCAT	-1.09889413	8.557399735	0.012787128	0.376829052
U3.24	-1.49142526	5.576968961	0.01283505	0.376829052
U6atac.29	-2.38352866	3.785989635	0.013059629	0.377523747
tRNA2-LeuTAA	-1.66936742	3.974107686	0.013474016	0.383601155
tRNA1-AsnGTT	-0.93147558	7.962088764	0.014090452	0.39410967
tRNA40-ThrAGT	-1.20252417	8.00746198	0.014483817	0.39410967
Y_RNA.684	2.380168702	3.849210771	0.014510103	0.39410967
snoU13.86	3.047260829	3.887450563	0.014700195	0.39410967
uc021qtn.1	-2.42558461	4.054546636	0.015164662	0.39410967
RNU6-9	-1.27226823	4.707463894	0.01590456	0.39410967
tRNA13-AlaTGC	-1.07694323	9.510390292	0.016125256	0.39410967
tRNA36-ArgACG	-1.42222279	6.510745574	0.016234233	0.39410967
Y_RNA.719	-1.87621292	4.049552581	0.016340695	0.39410967
RNU6-10	-1.35736219	4.333057065	0.016389921	0.39410967
SCARNA4	0.900162218	5.772147414	0.016691546	0.39410967
tRNA10-AlaCGC	-1.08617369	9.364295675	0.016698714	0.39410967
tRNA12-SerAGA	-2.17129695	3.960150632	0.016746309	0.39410967
uc011ley.2	-1.1603733	7.787151849	0.01677955	0.39410967
U1.116	-2.53057666	4.143384152	0.017351703	0.3975759
tRNA7-SerGCT	-1.22744148	8.048077286	0.017683906	0.3975759
U6.266	-1.68215765	4.162672228	0.018006863	0.3975759
tRNA136-AsnGTT	-1.18546894	6.252578135	0.018057145	0.3975759
uc031qaf.1	3.07982529	4.21672176	0.018100789	0.3975759
RNU4-9P	1.971224737	3.892961999	0.018196662	0.3975759
RNU6-48	-1.41494718	4.179417866	0.018610429	0.40194249
U4.64	-1.46842052	4.573943777	0.019052174	0.405290215
SNORA70.5	-2.3066839	3.705457206	0.019552672	0.405290215
tRNA83-AsnGTT	-0.95893311	7.650753844	0.019695599	0.405290215
tRNA21-ArgCCT	-1.3243702	9.463647642	0.019740822	0.405290215
RNU6-26	-1.26772628	4.674223977	0.019843906	0.405290215
tRNA4-AsnGTT	-0.95216219	7.630343605	0.020477143	0.409060595
U1.28	-1.66671858	4.666167958	0.020920233	0.409060595
U6.168	-1.75957771	3.988195609	0.021394709	0.409060595
RNU6-41	-1.2429829	4.599263654	0.021413125	0.409060595

tRNA15-ThrCGT	-0.94643604	8.011751983	0.021715336	0.409060595
tRNA88-PseudoCCT	1.406009411	4.775992477	0.021727046	0.409060595
SNORD116-19	-2.08108498	3.769046556	0.022072958	0.409060595
uc031qtw.1	-1.59586877	3.698957807	0.022111714	0.409060595
U6.422	-2.18510267	3.771206474	0.022136152	0.409060595
U2.3	-1.09052752	5.567176812	0.022205525	0.409060595
U3.20	-1.55295135	5.225744713	0.022487766	0.410237987
tRNA165-IleAAT	-0.81674152	8.525161575	0.023067622	0.416176914
snoU13.160	-1.88380693	3.690235583	0.023604159	0.416176914
tRNA36-ThrAGT	-1.09203025	7.99788872	0.023653935	0.416176914
tRNA6-TrpCCA	-1.06582891	10.59444161	0.023699271	0.416176914
SNORA57	-1.08263716	5.14915277	0.024108905	0.416665441
tRNA10-PseudoTGA	-1.42087364	5.662435588	0.024173287	0.416665441
uc001myu.3	-2.42408101	4.681561536	0.02449142	0.416665441
Y_RNA.428	-1.2545658	6.369684888	0.024614084	0.416665441
SCARNA1	-1.61719768	3.852134167	0.025631096	0.427567583
tRNA95-AlaAGC	-1.73027899	5.825175769	0.02592783	0.427567583
RNU6-27	-1.6140049	3.926299889	0.025940769	0.427567583
SNORA79.1	-1.15165538	4.196182085	0.026538748	0.433620064
tRNA9-IleAAT	-0.73350055	8.536774944	0.027547951	0.446033785
tRNA9-TyrGTA	-1.6487492	3.959996969	0.027773259	0.446033785
tRNA23-ArgCCG	-1.31033598	11.27930104	0.029044541	0.450180862
snoU13.318	-1.91587082	3.698261411	0.029045067	0.450180862
snoU13.328	2.684496203	4.280709032	0.029064976	0.450180862
RNU4-4P	-1.2311376	4.615863264	0.029137817	0.450180862
Y_RNA.633	-1.30999509	4.118055921	0.029229412	0.450180862
RNU5F-4P	-1.48557387	4.239223966	0.03016703	0.460844298
tRNA7-AsnGTT	-0.88097126	7.861805832	0.031177808	0.472444358
tRNA2-ArgCCT	-1.14460252	9.768619156	0.031689267	0.476353057
tRNA18-ArgCCT	-1.24884324	9.421599432	0.032541851	0.484021239
SNORD116-25	1.724675764	6.034751745	0.032861609	0.484021239
uc021ybm.1	-0.9249247	7.924193472	0.033450576	0.484021239
tRNA8-SerGCT	-0.98753133	8.589816708	0.033481121	0.484021239
tRNA11-IleAAT	-0.72451906	8.545784821	0.033487366	0.484021239
Y_RNA.597	1.732733959	3.760735382	0.034265836	0.491492414
snoU13.120	2.312826375	3.740373282	0.034550843	0.491826017
uc031qpu.1	2.324532842	3.768342356	0.035349099	0.49676233
RNU6-32	-1.18812391	4.327815738	0.035426372	0.49676233
tRNA100-PseudoGAA	-1.02843913	9.329302032	0.036035102	0.501555229
SNORD116-21	2.33201432	3.747426753	0.039146195	0.532853476
tRNA5-IleTAT	-1.08777911	7.207879458	0.039415342	0.532853476
Y_RNA.522	-1.81515534	4.101934504	0.039799275	0.532853476
tRNA12-ArgCCT	-1.27295081	9.216801318	0.039946538	0.532853476
U6atac.20	-1.94596283	4.036085302	0.040551585	0.532853476
RNU6-4	-1.52406279	4.105411071	0.040667896	0.532853476
SNORD3B-2	-0.79576357	8.540015612	0.040838684	0.532853476
tRNA11-AsnGTT	-1.30462038	4.970141103	0.040850618	0.532853476
tRNA31-AsnGTT	-0.87710624	7.603607761	0.041107699	0.532853476
Y_RNA.727	-1.25639747	4.115195415	0.041225337	0.532853476

RNU6-73	-1.46456929	4.007858615	0.042069664	0.532853476
SNORA65	-0.88817274	4.89950806	0.042220138	0.532853476
tRNA7-LeuAAG	-0.71492427	8.880350642	0.042319347	0.532853476
tRNA144-AspGTC	-0.82964134	11.54055138	0.042320413	0.532853476
U6atac.25	-1.77774451	3.762252674	0.042537531	0.532853476
tRNA1-ArgCCG	-0.76847131	7.276226466	0.043525106	0.535998445
SNORA7.4	-1.13362614	3.738511771	0.043665668	0.535998445
tRNA125-ThrCGT	-1.08045128	5.458664529	0.043842913	0.535998445
RNU6-45	-1.22266691	4.217769003	0.043929622	0.535998445
tRNA131-GlnCTG	-1.07262477	7.700551934	0.04461864	0.538855811
U6.1249	1.668615649	3.756761746	0.044737364	0.538855811
Y_RNA.245	2.25187274	3.754483152	0.045943642	0.545496798
U6.601	-1.35324491	3.970443965	0.046003943	0.545496798
RNU5E-4P	-1.0837447	5.498269856	0.046159655	0.545496798
tRNA66-AlaTGC	-0.81125784	9.226831102	0.046988475	0.545729532
tRNA32-MetCAT	-0.82679514	8.99856138	0.047086999	0.545729532
U4.79	-1.25353509	4.433132105	0.047164353	0.545729532
tRNA4-HisGTG	-1.61899083	3.790696889	0.047553836	0.545729532
U3.13	-1.51261982	3.797771351	0.047790048	0.545729532
RNU6-5	-1.02972321	4.777615387	0.048416122	0.545729532
SNORD36A	-1.75616298	3.84054929	0.048459133	0.545729532
tRNA1-LeuAAG	-0.6699966	8.883794181	0.048648388	0.545729532
RNU6-29	-1.13048434	4.36662749	0.048793274	0.545729532

Panel C

putative microRNAs	Sequences	logFC	logCPM	PValue	FDR
put-miR-718	TTCAGGTTACCGCAGG	-6.825255	9.009089	1.21E-05	0.01609
put-miR-594	TGATTTTTTTTGGCGAA	-8.122089	13.62184	4.24E-05	0.028197
put-miR-703	GACTGCTCGAGCTGCTT	-4.582706	9.340091	0.000177	0.065276
put-miR-477	TGGGCAGCAAGAATGG	-6.350308	8.65459	0.00021	0.065276
put-miR-727	GGAGGATTTCAATTGG	-4.944239	7.848316	0.000246	0.065276
put-miR-646	TCTGTCATGGCTGAGC	-6.551056	8.808979	0.000322	0.065276
put-miR-675	TACCTCAATTCTCTAGG	5.756095	8.531088	0.000387	0.065276
put-miR-319	TGTCGGCGGGGGCCCCGGAC	-3.557921	10.21937	0.000409	0.065276
put-miR-327	CATTCTCTGAAACTACA	-6.062301	8.473821	0.000441	0.065276
put-miR-338	GACTTGTGATTAGCGG	5.013978	10.59887	0.000571	0.067661
put-miR-1187	GAGCATGTTGACTGGAGA	-6.150807	8.514764	0.000634	0.067661
put-miR-6	GCGGACCTTGCTCAAGG	8.830503	11.10951	0.000659	0.067661
put-miR-341	TGAGAAAACATTTGAGG	-5.709943	8.260643	0.000661	0.067661
put-miR-750	CAAGAGATGAGGAATG	-5.585098	8.184314	0.000814	0.07204
put-miR-183	CCAGGCTGGTCGTGATGA	-5.716867	8.806919	0.000855	0.07204
put-miR-980	TCGGAAGAAGAAGCTGACCCA	-5.533531	8.158978	0.000866	0.07204
put-miR-798	TAACTCTTGGAAATCTTGG	-3.422593	9.962536	0.001016	0.075645
put-miR-765	AAAAAAGAGGGACAGAAATG	-5.173073	7.963608	0.001123	0.075645
put-miR-71	GACCTTGGGTTGGGTCGTGGT	-3.989796	8.732315	0.001131	0.075645
put-miR-1327	GAAAAGCAATCGTCACAG	-7.093524	9.887535	0.001158	0.075645
put-miR-371	AATATTCAGCAGTCAGACTGG	6.778311	9.768186	0.001224	0.075645
put-miR-354	CAGGGTGATGACTTCTG	-4.795128	7.781813	0.001302	0.075645
put-miR-688	GTGATGTCGGCTCATCGCAACCT	2.199185	10.66612	0.001307	0.075645
put-miR-756	TGAGGGATGTTTGGATGCTCGCTTTGA	2.032426	8.603553	0.001428	0.079215
put-miR-1146	CAACTGTAAGTCCATT	4.770832	9.196252	0.00158	0.079645
put-miR-1333	TAATTGTCCTCTTGGGG	-4.794112	7.786171	0.001619	0.079645
put-miR-511	AAGCAGGCGTTACAATG	-7.089632	9.54354	0.001631	0.079645
put-miR-1199	TGAACTCGGGAGAAGGAAGT	-2.994051	11.94621	0.001702	0.079645
put-miR-705	CCTGTCTGACTGGGTCTCC	6.097356	9.460857	0.00175	0.079645
put-miR-1275	AAGAAGTAGACTGGATTGG	-4.175643	7.541711	0.001817	0.079645
put-miR-1204	AGGGCTGGGCACGGGGG	-3.329219	8.43806	0.00195	0.079645
put-miR-857	TAAGTGGGAGTGCTCATG	5.519775	8.389269	0.001957	0.079645
put-miR-806	CACGAGAGAACGCACACC	7.044016	9.996928	0.002048	0.079645
put-miR-680	GTTAAGTATGCACAGG	6.207669	9.293799	0.002048	0.079645
put-miR-576	TGGCATCCTGTCTCTTGC	5.455074	8.34397	0.002094	0.079645
put-miR-444	CTGAAAAGGGGACGGATTGGGAA	-4.481465	8.254479	0.002327	0.083951

put-miR-1132	CACGCTGTCTTTTGTTTCTCT	-5.505609	11.5441	0.002357	0.083951
put-miR-1025	TGGAAGAGGGGAAAGGAGA	-4.492628	7.660657	0.002418	0.083951
put-miR-496	CTAAGAGTATGAGTAGC	8.320903	10.63469	0.00246	0.083951
put-miR-657	TCAGGACATTGGACTCT	-4.875422	9.301419	0.002633	0.087604
put-miR-625	GAGAAGACTGAATGCTCTTCTC	5.988262	8.702871	0.002699	0.087604
put-miR-40	GCTGGAATAGCTCAGTTGC	-4.339713	10.82143	0.00295	0.093474
put-miR-666	CAGCATCATGATCATTATGG	6.351541	8.967721	0.003077	0.095259
put-miR-1098	AAGGCGAAGGATATGTTG	7.117298	9.563651	0.003171	0.095916
put-miR-538	CATTGTGCTTCGTGGGAGAGTAGGGCA	-1.614431	8.767576	0.003422	0.101205
put-miR-952	GCCGACAGGTCCGGGTAA	-5.048736	10.09864	0.003556	0.101506
put-miR-572	TAGATTCAGGAACTGCCA	7.280517	9.715353	0.003598	0.101506
put-miR-972	TTTAGTAGAATTGTGTTGGGT	-3.277557	9.430308	0.003661	0.101506
put-miR-650	AAAAGCAAGGCGAAGAGG	-2.912473	9.278157	0.003775	0.102538
put-miR-134	CAGACTGCTCGAGCTGCTGC	-3.339267	9.218851	0.003866	0.102908
put-miR-975	TTTGCAGACTGGAAACT	5.625435	8.451316	0.004252	0.109356
put-miR-161	TGTCTGTAGCAATGTGCT	-6.214205	9.449224	0.004553	0.109356
put-miR-1265	TGGGGATAAAGTTAGGT	3.433066	12.55135	0.004559	0.109356
put-miR-968	TGGCTGTTGCACTTCT	8.03171	10.36384	0.004661	0.109356
put-miR-1125	TTGAAGACTGGCTCTCA	-4.530734	8.355286	0.00501	0.109356
put-miR-892	CAACGGTCTTGAAAACA	3.239723	8.81171	0.005015	0.109356
put-miR-1306	ATAACGTCATCTAGTGTG	-4.744221	10.56016	0.005065	0.109356
put-miR-948	TGGAAGAAAACGAGGAG	5.807023	8.569858	0.005204	0.109356
put-miR-925	ATTTTGGTCTGTTGGTT	4.384338	8.643659	0.005342	0.109356
put-miR-259	ATGTAACCGGGCTTTTGTGCT	-1.786601	8.598848	0.005378	0.109356
put-miR-209	GGTCCTCGGATCGGCC	-4.053427	9.542155	0.005417	0.109356
put-miR-773	CAGGCAGAAGGGAGCTTGT	-2.64537	7.45467	0.005514	0.109356
put-miR-910	CACGTTCTTCTCATGGT	-3.449747	7.34067	0.00564	0.109356
put-miR-664	AAAGTTGTGTTAGCTGA	5.719212	8.51182	0.005786	0.109356
put-miR-1068	TATGATTGTAAACTCTGA	5.890866	8.622725	0.005843	0.109356
put-miR-265	CACATGGGGGTAGAGCACTGACTGGG	2.110742	12.54113	0.005876	0.109356
put-miR-490	CTGCAGGAGTGTTTGAGA	2.057932	11.07639	0.005969	0.109356
put-miR-1155	CAAATGATCAAAGCAGG	2.494173	8.354137	0.006008	0.109356
put-miR-893	ACCATCCTCTGCTACCA	-2.575152	8.358309	0.006075	0.109356
put-miR-1342	ATCGGAAAATGTGGGAA	5.585468	8.418657	0.006226	0.109356
put-miR-967	AAATGCATTGGATATGG	7.085615	9.549591	0.006355	0.109356
put-miR-811	GTTGTAAAGCTCTGTTG	1.99226	9.063173	0.006362	0.109356
put-miR-812	GTTCTGAGTTCTTTGGTTGGA	7.523344	9.907158	0.006484	0.109356

put-miR-1084	AGGACCATTGCGTTGCC	6.454871	9.04042	0.006577	0.109356
put-miR-626	TTCACTACTTTATCTCTTTT	2.849801	9.706572	0.006577	0.109356
put-miR-127	AGACTGTGATGACTGGGAGAGCGGGCT	-2.251745	7.719415	0.006585	0.109356
put-miR-219	GAGGCTCGAGAGCAATGGC	4.972872	8.410708	0.006604	0.109356
put-miR-863	AAGTTGGAGTATGTTTTAGG	4.174184	8.270046	0.006626	0.109356
put-miR-33	CCATGACTGCAGATGG	-4.437318	8.062018	0.006671	0.109356
put-miR-1246	CTGGAGATCTGTTTGGGC	6.389535	8.993512	0.00675	0.109356
put-miR-126	GTGGGGCTTAGTGCTGA	-4.160086	8.05272	0.006849	0.109356
put-miR-459	GGGATCAACCTGACAA	7.00545	9.47012	0.00689	0.109356
put-miR-907	CCAACAGCTTCTGAGTTG	-5.599664	9.28021	0.006923	0.109356
put-miR-171	GTCCTGTGTCTTGTACGG	2.359948	10.86341	0.006942	0.109356
put-miR-1163	AGAGGACAGGGAAGCTT	6.420538	9.017069	0.006996	0.109356
put-miR-204	AGAAGTCAGAACCTCTAT	6.610111	9.154177	0.007138	0.109356
put-miR-498	GACAGATTTTAGCTTGTCC	2.214527	8.621153	0.007148	0.109356
put-miR-286	TAACAGGCGTTGAAATTGT	6.754071	9.266989	0.007468	0.112951
put-miR-223	GATGGGGGTAGAGCACTGC	2.25836	12.07399	0.007601	0.113671
put-miR-75	TGGGGATTGTGGGTTCTC	-2.009287	9.598075	0.00774	0.114174
put-miR-548	AGAGGACAGGGAAGCT	6.104866	8.784136	0.007923	0.114174
put-miR-1118	AGAGGGTTCCTGTAGACCTAGGGAGGA	-2.053777	10.22693	0.007925	0.114174
put-miR-943	AGCCAGAGTTCTGATTGTGAGTG	-4.954652	9.727141	0.007978	0.114174
put-miR-313	TGAGTTCTTTGGTCAGAA	6.693723	9.211486	0.00821	0.114289
put-miR-659	AGTAGCTGGTCGATTTGGC	6.996383	11.79431	0.008416	0.114289
put-miR-210	CTTCAGACTGTGAAACTGA	-2.997776	9.522779	0.008481	0.114289
put-miR-144	TAGAGATAGAGCTTATG	5.529267	8.376117	0.008489	0.114289
put-miR-563	TGGAGACATTAACTATGA	5.915791	8.643491	0.008604	0.114289
put-miR-1140	TGGGAAGGGCTGCCGG	-5.145788	9.310603	0.008606	0.114289
put-miR-497	TTGGTAGACTCTCACTT	-4.014605	9.04369	0.008664	0.114289
put-miR-573	TGGATAACTCTTTTTGTA	6.440895	9.021507	0.008788	0.114289
put-miR-906	TATGGGAAGAATCTGGG	6.054559	8.747883	0.008793	0.114289
put-miR-231	TATGTCATGGTGGCTTTGG	-2.9467	8.39848	0.008918	0.114289
put-miR-85	GAATCCCACTTCTGACACCA	-2.952626	7.663083	0.00901	0.114289
put-miR-884	ATGGTCTAGAGCTACAGGT	5.870477	8.628199	0.00911	0.114289
put-miR-918	CAGCTTCTTCCGCTTCTT	5.860051	8.60284	0.009174	0.114289
put-miR-985	GTACTGCATCTCTGCA	5.898607	8.628545	0.009188	0.114289
put-miR-320	ACTGGATCCAAGAAAAG	-2.920156	8.226536	0.009571	0.11795
put-miR-67	AGGACGTTGGTCAGAGC	-2.895467	7.636799	0.009857	0.119419
put-miR-221	TCTCTCAATCCTCTTGG	5.954163	8.658651	0.009869	0.119419

put-miR-471	AGAAGCAGAGAACGAGG	-3.987795	8.44957	0.009973	0.119585
put-miR-1352	TGGATTGTGGGGGAACC	5.478958	8.36409	0.010086	0.119857
put-miR-610	AGGAGATATAGCTCTTGT	5.722079	8.518132	0.010392	0.122408
put-miR-48	AAAATGGATTCGAACCA	5.632443	8.450136	0.010602	0.123785
put-miR-529	TGACCTTTTGCCTTCTGC	5.426419	8.338572	0.010802	0.125018
put-miR-424	TAAAAAGTTCTCTGTTTTTC	-2.78953	7.208748	0.010937	0.12549
put-miR-24	TTGGTGCATCTGTAGTCCAAC	-4.932824	10.24045	0.011113	0.126418
put-miR-164	TGAAATTCTAAATATTGCA	5.725561	8.51014	0.011372	0.126957
put-miR-229	TGGGATTTAGCTCAGC	3.799609	8.12666	0.011424	0.126957
put-miR-525	AAGTGGGAAGGCCCAGA	-3.723862	8.317629	0.011446	0.126957
put-miR-325	TTCAAATCCCACTTCTGACACCA	-2.927094	7.781125	0.012443	0.136691
put-miR-188	AAATGGCGATACTCAGG	6.335286	9.481014	0.012559	0.136691
put-miR-1066	TGCTTTGATCGTAGCCC	-3.205461	7.447675	0.012632	0.136691
put-miR-1295	GAGGTTAGGATATCTGGCT	-3.324069	7.580857	0.012893	0.13839
put-miR-588	TTTTGAACGTTCTTTCTT	1.985563	8.910298	0.013328	0.141683
put-miR-1273	AAGGGGAGGAATTTCACGTG	4.845324	8.579749	0.013413	0.141683
put-miR-783	AAGGAAAAAGCGGATA	-3.722555	7.559427	0.013621	0.14275
put-miR-37	TGGTGGAGTGAAGACG	-4.836261	8.547591	0.014174	0.14647
put-miR-1317	TGGAGTGTGGATTGGGG	1.625507	8.870365	0.014196	0.14647
put-miR-97	CTGCGTGGCTCTGACAC	3.27664	9.296956	0.014347	0.146889
put-miR-1016	AGGTAAAGCTCATGAGG	-2.141009	9.304303	0.014567	0.148003
put-miR-546	CCAAGGGGTTGTAGGGCCACT	3.880018	10.64815	0.015027	0.15152
put-miR-1099	AGACTCCTTTATCGTA	4.881265	8.359673	0.015178	0.151894
put-miR-874	ATGAGCATTGATTTAGG	-4.133563	8.481847	0.015742	0.15277
put-miR-701	TTGAGATTTGAGGGGGCCT	2.361165	8.446935	0.015782	0.15277
put-miR-279	CTGTGAAGCCTGTTGGTTTGCTGCTG	-2.002787	8.036423	0.015783	0.15277
put-miR-184	TGCTGAACCTCTGTATGT	5.624113	9.598097	0.015798	0.15277
put-miR-495	CAGGGAGTGAAAGAGAATT	-2.791411	9.973484	0.015923	0.15277
put-miR-90	GAAGTTAAATCCTTGGG	3.643929	8.886684	0.016047	0.15277
put-miR-468	ATAAGGAACTGCTCTCTC	4.936942	8.333361	0.016099	0.15277
put-miR-469	GCGGGGGATTAGCTCAGCTGGG	1.223156	14.10575	0.016186	0.15277
put-miR-1195	ATTCTTTGACATGCAGAT	2.561114	9.286221	0.016299	0.15277
put-miR-250	CTGGTGTAGAATTGAGG	-4.411037	8.166635	0.016733	0.153408
put-miR-1264	CAGGTTTCAGACTTTAGG	2.452989	9.271027	0.0168	0.153408
put-miR-465	CCAGTTGTCGTGGGTTTTT	2.030242	8.962972	0.016867	0.153408
put-miR-1243	TGTGGATTTTTGTCATGT	4.743252	8.639084	0.016871	0.153408
put-miR-771	CCTTGATCTGACTGGGGGCC	-1.886812	8.602159	0.016943	0.153408

put-miR-534	CTGTTGGAGAATTTGGAATATTAGGT	-4.34848	8.658536	0.01741	0.155563
put-miR-80	GAAGAGGGAGTGGTCTGTAAATGCG	6.380693	9.533096	0.017415	0.155563
put-miR-347	AAAGGGAAACAAGAATTCTT	1.937026	10.57284	0.017748	0.156927
put-miR-410	GGAGAATAGAACATGCTGATT	6.652715	9.666187	0.017824	0.156927
put-miR-891	TAAATGTTGGTTTGTTTGT	1.858836	8.369573	0.017921	0.156927
put-miR-189	GAGCGAAACGGCAGGAT	-2.368257	8.305643	0.018423	0.160266
put-miR-842	ACCCGGAGAACTGAACT	-2.045068	9.280359	0.01862	0.160514
put-miR-81	ATTTGAAAGAATGCTTG	2.468711	8.296721	0.018693	0.160514
put-miR-824	CTAGCTGAACCTCTGTAT	5.277761	8.805933	0.018935	0.161554
put-miR-958	ACAGGGCTGTGCAAAAA	3.475245	8.121044	0.019332	0.163891
put-miR-742	CAGGGCTGTGCTAACT	2.255055	7.794269	0.020414	0.171266
put-miR-605	TGAGCTTTGGAAGAAGGACCA	-2.303646	8.016644	0.020459	0.171266
put-miR-374	TGTGAGGATGTTCTGTAAGGAGTGTT	4.607626	8.644756	0.020848	0.173428
put-miR-135	TGAATTACGGAAGTGTTGGTTAAT	-3.311813	7.404022	0.021756	0.179859
put-miR-323	ATAAAATGGGCGTTGAGG	3.262325	8.71569	0.022489	0.184771
put-miR-506	AAGAGGGGCTTTTAGAACC	6.174059	11.22955	0.022884	0.186859
put-miR-293	GGGTAAAACTGCAGTGGGCGTTGGTAG	-1.800636	10.99339	0.02336	0.189584
put-miR-524	CGTGGTGATGCTCTGACA	-2.434924	7.973262	0.023561	0.189632
put-miR-517	TTGGGAAGGGCTGCCGGA	-4.206993	8.774926	0.023651	0.189632
put-miR-391	ATCTCGATCCAGTAGTC	1.801909	7.884667	0.024454	0.194897
put-miR-92	AGAGACTGACTTTGAGTA	5.755643	9.030306	0.025075	0.198658
put-miR-435	TTTAGACCGTTTTTATGTC	-3.735696	9.202301	0.027033	0.212767
put-miR-1080	TGTGGATTGATGCTCT	-1.707634	8.549845	0.027175	0.212767
put-miR-587	TTTGTAGAAGAGGAAGCG	-2.497802	8.421284	0.029781	0.231807
put-miR-1220	TAGAATGGGGCTTGTTGC	3.980805	8.446505	0.030296	0.233459
put-miR-1134	AAAGAATGAAGTTGGTCTGG	-1.684102	8.848135	0.030344	0.233459
put-miR-352	TAATGAATGACTGTTTG	1.774841	8.080119	0.030753	0.23524
put-miR-1162	AAAGAACGTTCAAAGG	2.807352	8.476403	0.030994	0.235731
put-miR-377	AAAGGGCTTTGACTATTT	1.681479	13.05617	0.031488	0.237918
put-miR-294	TGCTTCCTCAATCGGT	3.603279	8.466728	0.031639	0.237918
put-miR-1229	ACAGTAGCAATGTTCTGC	3.278472	7.88184	0.032091	0.238095
put-miR-1091	GATTATGATTGTGATTGTAGC	4.964642	9.493873	0.032144	0.238095
put-miR-419	AAAAAGTTAGACTTAGG	1.437457	9.015693	0.032199	0.238095
put-miR-249	GCCAGGATGTTGGCTTA	-1.617688	9.173125	0.03244	0.238442
put-miR-122	GGAACTCATGATTGTTGACTTTGG	-1.814811	8.353927	0.032687	0.238442
put-miR-1320	TTAGAGGCCACTCAAT	4.820365	8.331847	0.032994	0.238442
put-miR-335	TATTTAAATGAGAACTTTGAAGC	2.147361	9.043008	0.033061	0.238442

put-miR-166	CGTTTTGGTGTTGGTTG	-3.72297	8.052795	0.033142	0.238442
put-miR-1172	AGAACCAAGAAGCTCTGG	2.612818	8.384166	0.034733	0.246677
put-miR-1207	GTGAAAAGACATAGGGGG	1.882735	9.817827	0.034763	0.246677
put-miR-1130	TAGAAGAGGGAGCTTCTTT	2.429349	9.717761	0.034842	0.246677
put-miR-247	AGAGCTAGAATCCAGG	1.990242	8.786356	0.035469	0.249784
put-miR-246	TTTATTAGAGACGGGACTTT	4.838701	8.30995	0.035789	0.250379
put-miR-870	AAAGGATGTAGACAAGGGA	1.480442	8.130793	0.03593	0.250379
put-miR-704	CAAATGAATATCTGGGA	2.582114	8.282107	0.037366	0.258799
put-miR-266	TTTAGGTACTCTGAACAA	2.505244	8.581082	0.037586	0.258799
put-miR-928	CACAGAAGGAACGTTTAGA	2.539814	8.530779	0.037862	0.258799
put-miR-959	GAAAGAGAGTGAGACTCA	2.359451	8.451334	0.038062	0.258799
put-miR-676	TGGTGGTTGGTTTTGGG	-1.247728	9.504279	0.03818	0.258799
put-miR-1095	ATTGTGAATGATCTGG	4.006586	8.691739	0.038403	0.258799
put-miR-550	GCACTCTGGACTCTGAATCC	-1.460704	9.782298	0.038501	0.258799
put-miR-835	TTAGGAGTAGGTTACT	1.888177	8.674219	0.038693	0.258799
put-miR-686	TGACTGTTTTATATGAGTAA	1.693385	10.42287	0.038917	0.258993
put-miR-32	ATCCCGGACGAGCCCCCATTA	-1.878608	11.03059	0.039456	0.260368
put-miR-151	TTGAAACTGATATACTGTCTTAGG	-2.781416	7.308382	0.039515	0.260368
put-miR-726	GATGGGCTGATTGAGGGAT	3.117005	10.49059	0.039816	0.261058
put-miR-256	ATGAGGCTGAGATTGTCC	1.962535	8.272489	0.040595	0.264863
put-miR-368	AATGAGAACTTTGAAGGCCGAAG	2.363048	14.35762	0.041097	0.266314
put-miR-1232	ACAGACTGTCTTTGGG	2.165428	8.620526	0.041263	0.266314
put-miR-1219	GAAATTATTGATCCAGTCACGA	1.298803	9.720145	0.041418	0.266314
put-miR-1276	GAAGAGTTGATCCATG	-1.907277	9.309298	0.041682	0.266725
put-miR-1119	GTTTTGGGTTGAGTGAGGA	-2.455642	8.629875	0.042052	0.267807
put-miR-901	GTCTATTTGGATTATCGTC	1.728483	8.113002	0.042255	0.267816
put-miR-356	GTTTGAATTGGGAAGCTGGGGG	-1.750022	8.007003	0.043232	0.272707
put-miR-856	AAGGATAGGGAGGTATTT	2.182062	8.400665	0.044086	0.276787
put-miR-1244	CAGTTGGTAGAGCACCTGAC	-1.24774	10.46658	0.045019	0.281317
put-miR-476	GTGGTCAGGTAGAGAA	-1.919551	8.047207	0.046618	0.288241
put-miR-142	CAGTATAATTAGGGGTTAATTGTGGGA	1.249016	8.70991	0.046684	0.288241
put-miR-639	TGGAGTGTGGATTGGG	1.474835	8.945467	0.046777	0.288241
put-miR-622	TGAGTGGTCAATGGGGG	-1.336255	8.590784	0.047597	0.291931
put-miR-337	TGGGCTCAAGCTTTCTCT	-2.374139	8.27944	0.047999	0.291931
put-miR-422	TTTAGGGTTTTAAGGTGTTA	1.290525	11.98963	0.048034	0.291931
put-miR-1339	GGACTGAGAGCTCTTTCTG	3.091573	10.60449	0.048448	0.293113
put-miR-1356	GTGCTGCATGGCTGTCA	-1.345358	8.820822	0.049044	0.295237

put-miR-29	GAGATTTACGACCTAGG	1.337942	9.63023	0.049243	0.295237
put-miR-128	TGGCTCTGACACTAGTA	2.165955	8.272955	0.049525	0.295593
put-miR-359	GAAGTGGAAGGACTATGAA	2.152809	8.475685	0.050666	0.301056
put-miR-1003	TGGACTTCAGAACAGC	4.029771	7.874318	0.050924	0.301242
put-miR-238	AGAGACAGGAACTTTGATTTT	3.690637	8.383615	0.051316	0.302217
put-miR-961	TAGCTTGATCCAGTTG	2.181109	9.760789	0.052445	0.307507
put-miR-751	AGGCTGAGACTGGACAGAAAGACCT	-1.317479	8.022761	0.053486	0.312239
put-miR-1184	GCCTGCCCGGTGCTGGT	1.707305	8.999909	0.053825	0.312845
put-miR-379	GCGTGTGCGCGGGAGG	2.352869	7.584474	0.054246	0.313921
put-miR-361	CAGGTGAAATCGTGGATGT	2.224017	10.34793	0.05535	0.318896
put-miR-1111	TGATGCGTTGGGATGTAGC	-1.424105	8.543294	0.055717	0.318896
put-miR-938	TTAGGAAGCTGCTGATT	-1.317019	9.281869	0.055825	0.318896

eTable 2: List of 63 biomarkers validated in qPCR and used for statistical comparisons

Panel A (23 microRNAs)

microRNAs	Gene Number
hsa-let-7f-5p	MIMAT0000067
hsa-miR-1246	MIMAT0005898
hsa-miR-135b-5p	MIMAT0000758
hsa-miR-21-5p	MIMAT0000076
hsa-miR-425-5p	MIMAT0003393
hsa-miR-497-5p	MIMAT0002820
hsa-miR-148a-3p	MIMAT0000243
hsa-let-7a-5p	MIMAT0000062
hsa-let-7i-5p	MIMAT0000415
hsa-miR-143-3p	MIMAT0000435
hsa-miR-34b-3p	MIMAT0004676
hsa-miR-144-3p	MIMAT0000436
hsa-miR-16-1-3p	MIMAT0004489
hsa-miR-103a-3p	MIMAT0000101
hsa-miR-92a-3p	MIMAT0000092
hsa-let-7b-5p	MIMAT0000063

hsa-miR-142-5p	MIMAT0000433
hsa-miR-29c-3p	MIMAT0000433
has-miR-339-5p	MIMAT0000764
has-miR-107	MIMAT0000104
hsa-miR-126-3p	MIMAT0000445
hsa-miR-1271-5p	MIMAT0005796
hsa-miR-143-3p	MIMAT0000435

Panel B (29 small non-coding RNAs)

small non coding RNAs	Chr	Strand	Start	Stop	Sequence
RNU4-6p	X	-	16893269	16893390	TATCGTAGCCAATGAGGTTTATCCGAGGCGTGATTATTGCTAATTGAAAA
tRNA18ArgCCT	17	+	73030001	73030073	GCACTGGCCTCCTAAGCCAGGGATTGTGGGTTCGAGTCCCACCTGGGGTA
U6.428	1	+	180727858	180727953	AAGATTAGCATGAGGATGACACGCAAATTCGTGAAGCGTTCCATTTCTTT
RNU6-45	11	+	63737942	63738048	GGCCCTTGTGCAAGGATGACACGCAAATTCGTGAAGCGTTCCATATTTTT
RNU6-4	1	-	31970419	31970525	GGCCCCTGCACAGGGATGACACGCAAATTCGTGAAGCGTTCCATATTTTT
RNU6-6	2	+	201694732	201694839	GGCCCCTGTGCAAGGATGACACGCAAATTCGTGAAGCGTTCCATATTTTT
RNU6-7	3	+	194935516	194935622	GGCCCCTGCGCAAGGATGACATGCAAATTCGTGAAGCGTTCCATATTTTT
RNU6-73	13	+	28402900	28403006	GGCCCCTGTGCAAGGATGACATGCAAATTCGTGAAGCGTTCCATATTTTT
SNORD3B	17	+	18965225	18965440	CTTCTCTCCGTATTGGGGAGTGAGAGGGAGAGAACGCGGTCTGAGTGGTT
tRNA120-AlaAGC	6	-	28626014	28626085	GCGCATGCTTAGCATGCATGAGGTCCCGGGTTCGATCCCCAGCATCTCCA
tRNA73-ArgCCG	6	+	28849165	28849237	GCGTCTGATTCCGGATCAGAAGATTGAGGGTTCGAGTCCCTTCGTGGTCG
U6.168	6	-	18307204	18307310	ATGGCCCCTGCGCAAGGATGACACGCAAATTTGTGAAGGATTCCATATTT
U6.375	4	+	109573306	109573412	GGCCCCTGTGCAAGAATGACTCGCAAATTCGTGAAGCGTTCCATATTTTT
YRNA-684	18	+	20604559	20604666	GCTTCTTTTACTCTTTCCCTTCATTCTCACTACTGTACCTGATTCGTCTT
U6.601	19	-	39287642	39287749	GGCCCCTGCGCAAGGATGACATGCAAATTTGTGAAGTGTTCCATATTTTT
YRNA-255	17	+	80375102	80375197	GUGUCACCAACGUUGGUAUACAACCCCCCACAACUAAAUUUGACUGGCUU
tRNA9-TyrGTA	7	+	149255133	149255205	CTTTTTGACTGTAGAGCAAGAGGTCCCTGGTTCAAATCCAGGTTCTCCCT
U2.3	1	+	150209315	150209504	TCACTTCACGCATCGATCTGGTATTGCAGTACCTCCAGGAACAGTGCACC
U4.64	9	+	36267780	36267919	GTATCGTAGCCAATGAGGTTTATCCAAGGTGCGATTATTGCTAATTGAAA
SNORA57	10	+	27077946	27078086	TGCTGGCGGCTTCCCATCCGCTGGTTCTATCCTCAAACGCCGGGACACCG
UC022CJG1	Y	+	10037846	10037870	CATTGATCATCGACACTTCGAACGCACTTG
tRNA27-MetCAT	6	+	26766444	26766516	GCGTCAGTCTCATAATCTGAAGGTCCTGAGTTCGAGCCTCAGAGAGGGCA
tRNA8-ThrAGT	17	+	8090478	8090551	GCGCCTGTCTAGTAAACAGGAGATCCTGGGTTCGAATCCCAGCGGTGCCT
tRNA2-LeuTAA	4	-	156384978	156385052	GCATAAAACTTAAAATTTTATAATCAGAGGTTCAACTCCTCTTCTTAACA
YRNA-245	2	-	25919945	25920057	GTCTTTGTTGAACTCTTTCCCTCCTTCTCATTACTGTACTTGACCAGTCT
snoU13.120	4	+	17530560	17530663	GCTACCCTGGAACCTTGTTATGACATCTGCACATTACCCATCTGACCTGA
U6.1249	1	+	67661823	67661926	GATGGCATGACCCCTGATCAAGGACGGCATGCAAATTTGTGAAGTATTTC
tRNA84-GluTCC	1	-	1.61E+08	1.61E+08	TTTCACCGCCGCGGCCCGGGTTCGATTCCCGGTCAGGGAA
tRNA8-AlaTGC	12	+	1.25E+08	1.25E+08	TGCACGTATGAGGCCCCGGGTTCAATCCCCGGCATCTCCA

Panel C (11 putative microRNAs)

Putative miRs	Chr	Strand	Start	Stop	Sequence
put-miR-1204	4	+	1661740	1661756	AGGGCTGGGCACGGGGG
put-miR-1207	X	+	12647513	12647530	GTGAAAAGACATAGGGGG
put-miR-465	2	+	29475435	29475453	CCAGTTGTCGTGGGTTTTT
put-miR-6	16	+	67911172	67911188	GCGGACCTTGCTCAAGG

put-miR-325	3	+	193330821	193330843	TTCAAATCCCACTTCTGACACCA
put-miR-1146	5	+	67892184	67892199	ATAGGAGACTTCTATC
put-miR-742	16	+	49579869	49579884	CAGGGCTGTGCTAACT
put-miR-476	4	+	100244534	100244549	GTGGTCAGGTAGAGAA
put-mir-893	5	+	58317814	58317830	ACCATCCTCTGCTACCA
put-miR-594	17	+	46822348	46822364	TGATTTTTTTTGGCGAA
put-miR-1130	2	+	1.13E+08	1.13E+08	TAGAAGAGGGAGCTTCTTT

eTable 3: Univariate analysis of comparisons at time point T1

AUC, CI, count, ΔCq average, SD, ΔΔcq, fold change, t-test p value and power analysis for the comparison of individual biomarkers between HIA+ and HIA- in season 1. Samples were not collected from Uninjured and MSK groups at this time point.

HIA+ vs HIA-	AUC	95% Confidence interval	Count	Average Δcq HIA+	Average Δcq HIA-	SD HIA+	SD HIA-	ΔΔcq (HIA+) – (HIA-)	Fold change HIA+/ HIA-	t-test p-value Season 1	Power analysis (p = 0.05)
hsa-miR-103a-3p	0.66	0.53-0.78	43/32	-20.11	-20.45	0.57	0.65	0.34	1.27	0.023	177
hsa-miR-126-3p	0.71	0.58-0.84	37/28	-13.48	-14.56	1.19	1.89	1.08	2.11	0.012	108
hsa-miR-144-3p	0.67	0.54-0.79	40/30	-14.46	-16.19	2.24	3.12	1.73	3.32	0.008	124
hsa-miR-34b-3p	0.65	0.51-0.78	36/30	-13.93	-14.96	1.51	2.22	1.03	2.04	0.029	173
hsa-miR-92a-3p	0.66	0.53-0.78	42/33	-20.57	-20.86	0.59	0.55	0.29	1.22	0.039	304

eTable 4: Univariate analysis of comparisons at time point T2

AUC, CI, count, ΔCq average, SD, ΔΔcq, fold change, t-test p value and power analysis for each comparison of individual biomarkers between HIA+ and Uninjured, HIA- and MSK groups in season 1. T-test p values of season 2 are also reported when found ≤ 0.05

HIA+ vs Uninjured (U)	AUC	95% Confidence interval	Count	Average Δcq HIA+	Average Δcq U	SD HAI+	SD U	ΔΔcq (HIA+) - U	Fold change HIA+ / U	t-test p-value Season 1	Power analysis (p = 0.05)	t-test p-value Season 2
hsa-let-7f-5p	0.70	0.60-0.78	53/62	-16.24	-17.22	1.30	1.32	0.98	1.97	0.000	96	
hsa-let-7a-5p	0.70	0.59-0.77	53/62	-20.09	-20.36	0.49	0.37	0.27	1.21	0.001	139	0.031
hsa-let-7i-5p	0.65	0.55-0.73	53/62	-18.49	-18.72	0.42	0.49	0.23	1.17	0.010	234	
hsa-miR-135b-5p	0.68	0.58-0.76	50/61	-14.17	-14.80	0.90	0.97	0.63	1.55	0.001	122	
hsa-miR-34b-3p	0.75	0.66-0.83	34/45	-12.33	-13.99	2.02	2.46	1.66	3.16	0.002	104	0.046
RNU6-4	0.67	0.57-0.76	53/61	-19.90	-21.00	2.19	1.78	1.1	2.14	0.004	181	
RNU6-45	0.66	0.56-0.75	53/62	-18.86	-19.90	2.19	2.20	1.04	2.06	0.013	250	0.016
RNU6-6	0.65	0.56-0.74	53/61	-19.79	-20.73	2.00	1.79	0.94	1.92	0.009	228	0.051
RNU6-7	0.64	0.55-0.73	53/62	-19.82	-20.74	2.01	1.78	0.92	1.89	0.010	236	
RNU6-73	0.65	0.55-0.73	53/62	-19.76	-20.65	1.87	1.80	0.89	1.85	0.011	238	
SNORD3B-2	0.65	0.55-0.74	53/62	-19.25	-19.83	1.62	1.24	0.58	1.49	0.033	321	

YRNA-255	0.63	0.53-0.72	53/62	-22.71	-24.48	1.77	1.67	1.77	3.41	0.018	287	
hsa-miR-103a-3p	0.61	0.52-0.70	53/60	-19.70	-20.08	0.88	0.77	0.38	1.30	0.018	255	
U6.375	0.63	0.53-0.72	53/62	-16.38	-17.03	1.77	1.47	0.65	1.57	0.034	349	
U6.601	0.62	0.53-0.72	53/62	-18.17	-18.94	1.96	1.76	0.77	1.71	0.028	329	
YRNA-684	0.63	0.53-0.72	46/56	-12.30	-11.10	2.68	2.73	-1.2	0.44	0.028	254	
hsa-miR-107	0.59	0.49-0.68	53/62	-19.30	-19.61	0.93	0.73	0.31	1.24	0.047	150	
**HIA+ vs HIA-**	**AUC**	**95% Confidence interval**	**Count**	**Average Δcq HIA+**	**Average Δcq HIA-**	**SD HIA+**	**SD HIA-**	**ΔΔcq (HIA+) – (HIA-)**	**Fold change HIA+ / HIA-**	**t-test p-value Season 1**	**Power analysis (p = 0.05)**	**t-test p-value Season 2**
hsa-let-7a-5p	0.76	0.65-0.87	53/25	-20.09	-20.55	0.49	0.41	0.46	1.38	0.000	60	
hsa-let-7f-5p	0.89	0.82-0.96	53/25	-16.24	-17.90	1.29	0.56	1.66	3.16	0.000	26	
hsa-let-7i-5p	0.74	0.62-0.86	53/25	-18.49	-18.81	0.41	0.49	0.32	1.25	0.004	87	
hsa-miR-103a-3p	0.73	0.60-0.85	53/25	-19.70	-20.33	0.88	0.63	0.63	1.55	0.002	91	0.045
hsa-miR-107	0.71	0.59-0.83	53/25	-19.30	-19.80	0.93	0.52	0.5	1.41	0.014	141	
has-miR-1246	0.63	0.50-0.76	53/25	-21.39	-2079	1.33	1.02	-0.6	0.66	0.031	200	
has-miR-135b-5p	0.81	0.71-0.91	50/25	-14.17	-15.08	0.90	0.51	0.91	1.88	0.000	44	
hsa-miR-144-3p	0.69	0.56-0.82	42/24	-14.19	-15.87	2.60	2.73	1.68	3.20	0.016	108	
hsa-miR-34b-3p	0.71	0.56-0.86	34/18	-12.33	-13.84	2.02	2.22	1.51	2.85	0.016	102	
hsa-miR-92a-3p	0.74	0.62-0.85	52/25	-20.38	-20.76	0.50	0.57	0.38	1.30	0.004	90	
put-miR-476	0.68	0.55-0.81	52/24	-16.19	-17.87	3.03	2.39	1.68	3.20	0.020	323	
put-miR-6	0.64	0.52-0.77	51/24	-14.42	-16.13	3.49	2.65	1.71	3.27	0.037	85	0.006
RNU6-4	0.73	0.67-0.88	52/25	-19.90	-21.80	2.19	1.65	1.9	3.73	0.000	62	
RNU6-45	0.76	0.65-0.86	52/24	-18.86	-20.85	2.19	1.72	1.99	3.97	0.000	59	0.003
RNU6-6	0.76	0.66-0.87	53/25	-19.78	-21.56	2.01	1.63	1.78	3.43	0.000	61	0.006
RNU6-7	0.75	0.65-0.86	53/25	-19.82	-21.54	2.01	1.69	1.72	3.29	0.000	66	0.043
RNU6-73	0.75	0.64-0.86	53/25	-19.76	-21.39	1.87	1.74	1.63	3.10	0.000	66	0.050
SNORD3B-2	0.66	0.54-0.79	53/25	-19.25	-20.07	1.62	1.07	0.82	1.77	0.025	186	0.038
tRNA120-AlaAGC	0.64	0.52-0.77	51/24	-16.29	-14.34	3.65	2.67	-1.95	0.26	0.023	98	
tRNA18-ArgCCT	0.72	0.61-0.84	53/25	-22.31	-23.74	1.80	1.67	1.43	2.69	0.001	73	
tRNA27-MetCAT	0.68	0.56-0.80	52/25	-18.36	-16.86	1.88	1.31	-1.27	0.41	0.030	176	
tRNA73-ArgCCG	0.68	0.56-0.80	52/25	-16.65	-18.12	2.31	1.72	1.47	2.77	0.006	66	
U2.3	0.64	0.51-0.76	53/25	-26.58	-27.64	2.13	0.96	1.06	2.08	0.003	162	

U6.375	0.71	0.59-0.83	53/25	-16.38	-17.70	1.77	1.58	1.32	2.50	0.002	92	0.005
U6.601	0.72	0.61-0.84	53/25	-18.17	-19.73	1.96	1.75	1.56	2.95	0.001	77	
Y_RNA.255	0.70	0.58-0.81	53/25	-23.71	-24.94	1.77	1.40	1.23	2.35	0.003	93	0.038

**HIA+ vs MSK**	**AUC**	**95% Confidence Interval**	**Count**	**Average Δcq HIA+**	**Average Δcq MSK**	**SD HIA+**	**SD MSK**	**ΔΔcq (HIA+)-MSK**	**Fold change HIA+/ MSK**	**t-test p-value Season 1**	**Power analysis (p = 0.05)**	**t-test p-value Season 2**
tRNA18ArgCCT	0.73	0.62-0.83	53/31	-22.31	-23.57	1.80	1.45	1.26	2.39	0.001	123	
RNU6-7	0.69	0.57-0.81	53/31	-19.82	-21.11	2.01	1.77	1.29	2.45	0.004	322	0.007
U6.168	0.69	0.57-0.81	52/30	-16.74	-17.78	1.67	1.62	1.04	2.06	0.008	335	0.007
hsa-miR-143-3p	0.68	0.56-0.81	53/31	-18.17	-17.64	0.92	1.03	-0.53	0.69	0.017	128	0.031
RNU6-45	0.69	0.57-0.81	53/31	-18.86	-20.32	2.19	2.21	1.46	2.75	0.004	290	0.001
RNU6-4	0.69	0.57-0.86	53/31	-19.90	-21.20	2.19	2.09	1.3	2.46	0.009	396	
RNU6-73	0.69	0.57-0.82	53/31	-19.76	-20.93	1.87	2.01	1.17	2.25	0.009	458	0.002
U6.375	0.67	0.55-0.79	53/30	-16.38	-17.39	1.77	1.50	1.01	2.01	0.010	433	0.002
hsa-miR-16-1-3p	0.68	0.56-0.79	53/31	-14.16	-14.94	1.41	1.36	0.78	1.72	0.016	166	0.000
YRNA-255	0.67	0.55-0.79	53/31	-23.71	-24.77	1.77	1.67	1.06	2.08	0.008	260	0.008
RNU6-6	0.69	0.57-0.81	53/31	-19.79	-20.98	2.01	2.10	1.19	2.28	0.011	499	0.001
UC022CJG1	0.66	0.54-0.78	53/31	-22.05	-22.65	1.05	0.99	0.6	1.52	0.011	127	
SNORA57	0.63	0.50-0.76	53/31	-22.82	-21.34	1.15	1.13	-1.48	0.36	0.050	516	0.015
U6.601	0.69	0.56-0.81	53/31	-18.17	-19.21	1.96	2.07	1.04	2.06	0.024	231	0.005

eTable 5: Univariate analysis of comparisons at time point T3

AUC, CI, count, ΔCq average, SD, ΔΔcq, fold change, t-test p value and power analysis for each comparison of individual biomarkers between HIA+ and Uninjured, HIA- and MSK groups in season 1. T-test p values of season 2 are also reported when found ≤ 0.05.

**HIA+ vs Uninjured (U)**	**AUC**	**95% Confidence interval**	**Count**	**Average Δcq HIA+**	**Average Δcq U**	**SD HAI+**	**SD U**	**ΔΔcq (HIA+) - U**	**Fold change HIA+/ U**	**t-test p-value Season 1**	**Power analysis (p = 0.05)**	**t-test p-value Season 2**
hsa-miR-144-3p	0.72	0.62-0.80	44/39	-12.54	-13.94	1.94	2.50	1.4	2.64	0.005	133	
hsa-let-7f-5p	0.63	0.52-0.72	55/46	-16.33	-16.95	1.44	1.41	0.62	1.54	0.033	279	0.007
U6.1249	0.65	0.56-0.74	41/32	-10.16	-8.89	3.17	2.30	-1.27	0.41	0.051	221	

HIA+ vs HIA-	AUC	95% Confidence interval	Count	Average Δcq HIA+	Average Δcq HIA-	SD HIA+	SD HIA-	ΔΔcq (HIA+) – (HIA-)	Fold change HIA+/ HIA-	t-test p-value Season 1	Power analysis (p = 0.05)	t-test p-value Season 2
hsa-let-7a-5p	0.69	0.52-0.82	55/20	-20.33	-20.62	0.56	0.43	0.29	1.22	0.042	154	
hsa-let-7f-5p	0.89	0.82-0.96	55/20	-16.33	-18.16	1.44	0.65	1.83	3.56	0.000	28	0.007
hsa-miR-103a-3p	0.74	0.61-0.86	54/20	-19.71	-20.38	0.84	0.65	0.67	1.59	0.002	75	0.001
hsa-miR-107	0.73	0.60-0.86	55/20	-19.31	-19.85	0.59	0.81	0.54	1.45	0.008	107	0.037
hsa-miR-144-3p	0.69	0.55-0.83	44/20	-12.54	-14.01	1.94	2.44	1.47	2.77	0.012	161	0.000
hsa-miR-148a-3p	0.66	0.51-0.80	55/20	-19.09	-19.62	1.11	0.55	0.53	1.44	0.007	212	
hsa-miR-21-5p	0.71	0.56-0.85	55/20	-21.88	-22.55	0.92	0.87	0.67	1.59	0.006	89	0.005
hsa-miR-34b-3p	0.70	0.54-0.86	41/12	-12.34	-13.99	2.30	2.16	1.65	3.14	0.032	98	0.000
put-miR-465	0.77	0.64-0.89	39/18	-13.15	-10.84	2.59	2.13	-2.31	0.20	0.002	1666	
RNU6-4	0.70	0.57-0.83	55/20	-20.26	-21.46	1.88	1.42	1.2	2.30	0.012	121	
RNU6-45	0.69	0.56-0.82	55/20	-19.22	-20.51	2.05	1.45	1.29	2.45	0.011	123	
RNU6-6	0.69	0.56-0.82	55/20	-20.09	-21.24	1.81	1.43	1.15	2.22	0.012	123	0.012
RNU6-7	0.68	0.54-0.81	55/20	-20.13	-21.17	1.79	1.47	1.04	2.06	0.023	153	0.033
RNU6-73	0.67	0.54-0.81	55/20	-20.02	-21.08	1.80	1.47	1.06	2.08	0.021	149	
tRNA2-LeuTAA	0.60	0.47-0.73	55/20	-17.03	-17.63	1.56	0.91	0.6	1.52	0.049	248	
U2.3	0.64	0.50-0.77	55/20	-26.57	-27.52	1.96	1.06	0.95	1.93	0.042	180	
U6.601	0.66	0.53-0.79	54/20	-18.42	-19.46	1.89	1.60	1.04	2.06	0.034	177	
snoU13.120	0.60	0.52-0.82	32/16	-12.59	-12.95	2.46	2.11	0.36	1.28	0.05	228	0.018

HIA+ vs MSK	AUC	95% Confidence Interval	Count	Average Δcq HIA+	Average Δcq MSK	SD HIA+	SD MSK	ΔΔcq (HIA+) - MSK	Fold change HIA+ / MSK	t-test p-value Season 1	Power analysis (p = 0.05)	t-test p-value Season 2
hsa-let-7f-5p	0.71		55/25	-20.33	-20.28	0.56	1.06	-0.05	0.97	0.011	107	

		0.59-0.83										
hsa-miR-135b-5p	0.73	0.61-0.86	52/25	-14.95	-14.08	0.96	1.30	-0.87	0.55	0.001	72	0.019
hsa-miR-425-5p	0.71	0.53-0.85	55/25	-18.47	-19.20	0.87	1.09	0.73	1.66	0.002	134	
hsa-miR-16-1-3p	0.74	0.63-0.86	54/25	-13.80	-15.06	1.59	1.33	1.26	2.39	0.001	75	0.000
U6.428	0.71	0.58-0.83	46/21	-13.09	-14.26	1.53	1.59	1.17	2.25	0.006	147	0.012
hsa-miR-144-3p	0.80	0.69-0.91	44/15	-12.54	-14.57	1.94	2.49	2.03	4.08	0.002	75	0.016
YRNA-255	0.70	0.58-0.82	55/25	-23.91	-25.06	1.71	1.47	1.15	2.22	0.006	292	0.008
SNORA57	0.68	0.55-0.81	55/25	-20.64	-21.42	1.20	1.05	0.78	1.72	0.006	159	0.015
hsa-miR-21-5p	0.67	0.53-0.80	55/25	-21.84	-21.40	0.92	1.03	-0.44	0.74	0.039	151	
put-miR-893	0.66	0.53-0.79	55/25	-27.77	-29.07	2.69	2.28	1.3	2.46	0.039	181	
tRNA27-MetCAT	0.65	0.53-0.78	55/25	-18.53	-19.42	1.76	1.53	0.89	1.85	0.032	346	0.016
tRNA18ArgCCT	0.64	0.52-0.77	55/25	-22.59	-23.74	2.16	1.60	1.15	2.22	0.020	358	
tRNA8-ThrAGT	0.64	0.51-0.76	55/25	-18.08	-19.24	2.82	2.10	1.16	2.23	0.045	2778	
RNU4-6P	0.61	0.47-0.74	55/25	-16.98	-16.23	1.49	1.09	-0.75	0.59	0.027	173	
U6.168	0.64	0.49-0.78	55/25	-16.78	-17.60	1.57	1.92	0.82	1.77	0.046	619	0.007

eTable 6: Pairwise comparison of HIA+ and baseline values

Count, ΔCq average, SD, ΔΔcq, fold change and paired-sample test for HIA+ values recorded in season 1 at each time point and pre-season baseline.

HIA+ (T1) vs Baseline	Count	Average Δcq HIA+ (T1)	Average Δcq B	SD HIA+ (T1)	SD B	ΔΔcq HIA+ (*a*) - B	Fold change HIA+ (*a*) / B	Pair sample test Sig. (2-tailed)
hsa-let-7f-5p	219	-18.01	-17.67	0.74	1.27	-0.35	-1.27	0.02
hsa-miR-1246	219	-21.66	-20.79	0.75	1.04	-0.87	-1.83	0.00

put-miR-1207	207	-15.59	-12.28	3.27	2.22	-3.30	-9.88	0.00
RNU4-6p	219	-17.44	-16.85	1.12	1.09	-0.59	-1.51	0.00
put-miR-468	64	-14.10	-12.25	1.33	1.70	-1.85	-3.60	0.05
has-miR-148a-3p	219	-19.97	-19.67	0.88	0.66	-0.29	-1.22	0.01
hsa-miR-143-3p	114	-18.00	-18.17	0.99	0.92	0.16	1.12	0.00
hsa-miR-34b-3p	182	-13.93	-12.33	1.51	1.87	-1.60	-3.04	0.00
U2.3	219	-27.18	-27.13	1.32	1.31	-0.05	-1.03	0.01
U4.64	107	-11.33	-11.90	2.36	2.64	0.57	1.49	0.00
tRNA27-MetCAT	112	-18.60	-18.36	1.48	1.88	-0.24	-1.18	0.01
hsa-let-7b-5p	219	-20.07	-20.26	0.58	0.58	0.19	1.14	0.00
hsa-miR-142-3p	114	-23.48	-23.56	0.77	0.87	0.08	1.06	0.00
hsa-miR-142-5p	219	-20.83	-20.14	0.90	1.03	-0.69	-1.61	0.00
hsa-miR-29c-3p	219	-19.93	-19.74	0.58	0.58	-0.19	-1.14	0.00
put-miR-1146	122	-12.59	-11.17	2.39	2.97	-1.43	-2.69	0.00
tRNA2-LeuTAA	219	-16.87	-17.65	1.30	1.47	0.78	1.71	0.01
tRNA9-TyrGTA	206	-13.04	-11.65	2.42	2.07	-1.39	-2.61	0.00
YRNA-245	219	-15.50	-14.70	1.42	1.36	-0.80	-1.74	0.01
**HIA+ (T2) vs B**	**Count**	**Average Δcq HIA+ (T2)**	**Average Δcq B**	**SD HIA+ (T2)**	**SD B**	**ΔΔcq HIA+ (T2) - B**	**Fold change HIA+ (T2) / B**	**Pair sample test Sig. (2-tailed)**
hsa-let-7f-5p	229	-16.24	-17.67	1.30	1.27	1.43	2.69	0.01
hsa-miR-1246	229	-21.39	-20.79	1.33	1.04	-0.60	-1.52	0.01
hsa-miR-135b-5p	226	-14.17	-15.45	0.90	0.96	1.28	2.43	0.00
hsa-miR-21-5p	229	-21.86	-22.45	0.99	0.74	0.59	1.51	0.03
hsa-miR-425-5p	229	-18.87	-18.37	0.71	0.86	-0.50	-1.41	0.00
put-miR-1207	217	-13.48	-12.28	3.36	2.22	-1.20	-2.30	0.04
hsa-let-7a-5p	229	-20.09	-20.65	0.49	0.41	0.56	1.47	0.00
hsa-miR-143-3p	229	-18.17	-17.60	0.92	1.18	-0.57	-1.48	0.00
hsa-miR-34b-3p	180	-12.33	-12.33	2.02	1.87	0.00	-1.00	0.03
SNORD3B	229	-19.25	-20.20	1.62	1.03	0.94	1.92	0.02
U6.168	228	-16.74	-16.82	1.67	1.56	0.07	1.05	0.03
YRNA-684	196	-12.30	-10.58	2.68	2.12	-1.72	-3.29	0.00
hsa-miR-144-3p	208	-14.19	-13.70	2.60	2.06	-0.49	-1.41	0.01
hsa-miR-16-1-3p	226	-14.16	-13.71	1.41	1.49	-0.45	-1.36	0.03
put-miR-893	229	-28.04	-27.27	2.65	1.94	-0.77	-1.71	0.04
U4.64	221	-11.90	-10.76	2.64	1.89	-1.14	-2.20	0.00
SNORA57	229	-20.82	-20.63	1.15	1.06	-0.20	-1.15	0.04
UC022CJG1	229	-22.04	-22.64	1.05	0.94	0.60	1.51	0.02
hsa-let-7b-5p	229	-20.21	-20.26	0.90	0.58	0.05	1.04	0.01
hsa-miR-142-3p	229	-23.56	-22.94	0.87	1.11	-0.63	-1.55	0.00
hsa-miR-142-5p	229	-20.82	-20.14	0.97	1.03	-0.68	-1.61	0.00
hsa-miR-29c-3p	229	-19.79	-19.74	0.90	0.58	-0.05	-1.04	0.01

tRNA2-LeuTAA	227	-16.87	-17.65	1.99	1.47	0.78	1.72	0.02
tRNA9-TyrGTA	213	-13.06	-11.65	2.84	2.07	-1.41	-2.65	0.00
YRNA-245	229	-15.18	-14.70	1.83	1.36	-0.48	-1.40	0.00
hsa-miR-339-5p	229	-15.89	-15.50	1.60	1.30	-0.39	-1.31	0.02
**HIA+ (T3) vs B**	**Count**	**Average Δcq HIA+ (T3)**	**Average Δcq B**	**SD HIA+ (T3)**	**SD B**	**ΔΔcq HIA+ (T3) - B**	**Fold change HIA+ (T3) / B**	**Pair sample test Sig. (2-tailed)**
hsa-let-7f-5p	231	-16.33	-17.67	1.44	1.27	1.34	2.52	0.02
hsa-miR-1246	231	-21.19	-20.79	1.21	1.04	-0.40	-1.32	0.01
hsa-miR-135b-5p	228	-14.95	-15.45	0.96	0.96	0.50	1.41	0.00
hsa-miR-21-5p	231	-21.88	-22.45	0.92	0.74	0.57	1.48	0.01
U6.428	215	-13.10	-12.76	1.53	1.69	-0.34	-1.26	0.02
U6.168	231	-16.78	-16.82	1.57	1.56	0.04	1.03	0.04
YRNA-684	201	-11.24	-10.58	3.03	2.12	-0.66	-1.58	0.02
hsa-miR-92a-3p	231	-20.47	-20.50	0.68	0.46	0.03	1.02	0.04
U4.64	231	-16.98	-16.85	1.49	1.09	-0.13	-1.09	0.02
U6.1249	160	-10.16	-8.96	3.17	2.18	-1.20	-2.30	0.02
RNU4-6P	231	-16.98	-16.85	1.49	1.08	-0.13	-1.09	0.03

eTable 7: Multivariable logistic regression analysis of concussion biomarkers across season-1 (training dataset) and season-2 (test dataset)

AUCs (95% CI) of biomarker panel in seasons 1 (training dataset) and season 2 (test dataset) across different comparisons and time points. Fourteen biomarkers were included in this model. Under all control groups, HIA-, uninjured and MSK controls are included. P values are shown underneath each comparison.

HIA+ (CONCUSSED) vs.					
	**All control groups**	**HIA-**	**Uninjured**	**MSK controls**	**Baselines**
SEASON 1 (2017-18)					
Time point T1	-	**1 (1-1)**	**-**	**-**	**1 (1-1)**
p value		0.000			0.000
Time point T2	**0.91 (0.81-1.00)**	**0.88 (0.74-1.00)**	**0.93 (0.84-1.00)**	**0.90 (0.78-1.00)**	**0.95 (0.89-1.00)**
p value	0.000	0.006	0.000	0.000	0.000
Time point T3	**0.94 (0.86-1.00)**	**0.96 (0.89-1.00)**	**0.96 (0.88-1.00)**	**0.90 (0.69-1.00)**	**0.91 (0.84-0.98)**
p value	0.000	0.000	0.000	0.005	0.000

SEASON 2 (2018-19)					
Time point T2	**0.96**<br>**(0.92-1.00)**	**0.94**<br>**(0.85-1.00)**	**0.94**<br>**(0.87-1.00)**	**1.00**<br>**(1.00-1.00)**	**-**
p value	0.000	0.000	0.000	0.000	
Time point T3	**0.93**<br>**(0.86-1.00)**	**0.86**<br>**(0.73-1.00)**	**0.95**<br>**(0.89-1.00)**	**0.95**<br>**(0.88-1.00)**	**-**
p value	0.000	0.000	0.000	0.000	

eTable 8: Summary statistics of the 14 biomarkers

**has-let-7a-5p**	**Mean**	**SD**	**Min**	**Median**	**Max**	**IQR**	**CV**
**Baseline**	-20.56	0.41	-22.09	-20.66	-19.59	0.48	-0.02
**HIA+ T1**	-20.46	0.51	-21.69	-20.38	-19.15	0.56	-0.02
**HIA+ T2**	-20.09	0.49	-20.95	-20.06	-28.86	0.57	-0.02
**HIA+ T3**	-20.33	0.56	-22.33	-20.32	-19.08	0.65	-0.03
**HIA- T1**	-20.61	0.44	-21.55	-20.55	-19.89	0.57	-0.02
**HIA- T2**	-20.55	0.41	-21.56	-20.51	-19.55	0.44	-0.02
**HIA- T3**	-20.62	0.43	-21.51	-20.65	-19.53	0.37	-0.02
**Uninjured T2**	-20.36	0.37	-21.18	-20.35	-19.65	0.47	-0.02
**Uninjured T3**	-20.49	0.61	-21.63	-20.52	-18.14	0.75	-0.03
**MSK T2**	-20.26	0.72	-21.23	-20.52	-18.29	0.65	-0.04
**MSK T3**	-20.38	0.62	-21.22	-20.22	-18.74	0.85	-0.03

**has-miR-103a-3p**	**Mean**	**SD**	**Min**	**Median**	**Max**	**IQR**	**CV**
**Baseline**	-20.16	0.61	-22	-20.19	-17.47	0.69	-0.03
**HIA+ T1**	-20.11	0.58	-21.02	-20.18	-18.12	0.62	-0.03
**HIA+ T2**	-19.7	0.88	-21.14	-19.81	-16.92	0.79	-0.04
**HIA+ T3**	-19.71	0.84	-20.76	-19.92	-16.4	0.85	-0.04
**HIA- T1**	-20.45	0.65	-22.27	-20.38	-18.87	0.62	-0.03
**HIA- T2**	-20.33	0.63	-22.01	-20.28	-18.73	0.66	-0.03
**HIA- T3**	-20.38	0.65	-22.21	-20.31	-18.98	0.39	-0.03

**Uninjured T2**	-20.08	0.77	-21.88	-20.17	-17.85	0.91	-0.04
**Uninjured T3**	-19.87	0.88	-21.15	-19.99	-17.27	0.71	-0.04
**MSK T2**	-19.82	0.79	-21.45	-19.83	-17.52	0.78	-0.04
**MSK T3**	-19.61	0.68	-21.09	-19.68	-18	0.89	-0.031

**has-miR-143-3p**	**Mean**	**SD**	**Min**	**Median**	**Max**	**IQR**	**CV**
**Baseline**	-17.6	1.81	-20.95	-17.77	-13.9	1.62	-0.07
**HIA+ T1**	-18.03	0.85	-19.43	-18.1	-15.7	1.25	-0.05
**HIA+ T2**	-18.17	0.92	-19.58	-18.36	-15.74	1.27	-0.05
**HIA+ T3**	-17.56	1.08	-19.4	-17.78	-15.25	1.32	-0.06
**HIA- T1**	-18.14	0.79	-19.78	-18.23	-16.36	1.19	-0.04
**HIA- T2**	-17.93	0.82	-19.52	-17.91	-16.67	1.23	-0.05
**HIA- T3**	-17.91	1.02	-19.7	-17.99	-15.74	1.28	-0.06
**Uninjured T2**	-18	0.99	-19.91	-18.01	-16.03	1.29	-0.05
**Uninjured T3**	-17.68	1.01	-19.5	-17.48	-16.06	1.63	-0.06
**MSK T2**	-17.46	1.03	-19.7	-17.57	-16.18	1.24	-0.06
**MSK T3**	-17.01	1.25	-19.59	-17.32	-14.59	1.75	-0.07

**has-miR-34b-3p**	**Mean**	**SD**	**Min**	**Median**	**Max**	**IQR**	**CV**
**Baseline**	-12.33	1.87	-17.73	-12.04	-8.45	2.44	-0.15
**HIA+ T1**	-13.93	1.51	-17.12	-14.06	-10.29	2.3	-0.11
**HIA+ T2**	-12.33	2.02	-16.98	-12.18	-8.71	2.47	-0.16
**HIA+ T3**	-12.34	2.3	-16.82	-11.69	-7.47	3.4	-0.19
**HIA- T1**	-14.96	2.22	-20.24	-14.85	-10.44	2.37	-0.15
**HIA- T2**	-13.84	2.22	-18.91	-13.63	-9.58	1.76	-0.16
**HIA- T3**	-13.99	2.16	-17.67	-13.71	-10.4	1.89	-0.15
**Uninjured T2**	-13.97	2.48	-19.2	-13.85	-9.21	3.41	-0.18
**Uninjured T3**	-13.04	2.14	-19.39	-13.04	-8.9	2.81	-0.16
**MSK T2**	-12.84	1.58	-15.19	-12.66	-9.71	2.65	-0.12
**MSK T3**	-12.25	2.03	-15.92	-12.14	-8.63	2.1	-0.17

**RNU6.45**	**Mean**	**SD**	**Min**	**Median**	**Max**	**IQR**	**CV**

**Baseline**	-19.96	1.72	-23.6	-20.1	-14.29	2.29	-0.09
**HIA+ T1**	-20.04	1.91	-23.82	-19.88	-14.64	2.15	-0.1
**HIA+ T2**	-18.86	2.19	-24.09	-18.92	-11.97	2.82	-0.12
**HIA+ T3**	-19.22	2.05	-23.87	-19.05	-14.69	1.85	-0.11
**HIA- T1**	-20.18	1.74	-23.27	-19.94	-16.78	2.55	-0.09
**HIA- T2**	-20.81	1.73	-23.7	-20.06	-18.29	2.33	-0.08
**HIA- T3**	-20.51	1.45	-22.87	-20.72	-18.38	2.33	-0.07
**Uninjured T2**	-19.86	2.2	-23.61	-20	-13.53	2.95	-0.11
**Uninjured T3**	-19.65	2.39	-23.74	-19.92	-13	2.94	-0.12
**MSK T2**	-20.32	2.21	-24.03	-20.64	-14.45	2.88	-0.11
**MSK T3**	-20.14	2.45	-23.49	-20.32	-12.75	2.86	-0.12

**RNU6.7**	**Mean**	**SD**	**Min**	**Median**	**Max**	**IQR**	**CV**
**Baseline**	-20.6	1.67	-24.25	-20.76	-15.07	2.22	-0.08
**HIA+ T1**	-20.81	1.67	-24.4	-20.4	-17.9	2.06	-0.08
**HIA+ T2**	-19.82	2.01	-24.56	-19.83	-13.14	2.2	-0.1
**HIA+ T3**	-20.13	1.79	-24.49	-19.72	-16.32	1.69	-0.09
**HIA- T1**	-20.9	1.59	-23.89	-20.46	-17.33	2.22	-0.08
**HIA- T2**	-21.54	1.69	-24.42	-20.77	-19.18	2.72	-0.08
**HIA- T3**	-21.17	1.47	-23.75	-21.4	-18.89	2.34	-0.07
**Uninjured T2**	-20.72	1.78	-24.54	-20.76	-16.84	2.5	-0.09
**Uninjured T3**	-20.45	2.01	-24.23	-20.63	-14.71	2.56	-0.1
**MSK T2**	-21.11	1.77	-24.29	-21.11	-17.3	2.35	-0.08
**MSK T3**	-20.96	2.11	-23.94	-21.27	-16.21	3.14	-0.1

**snoU13.120**	**Mean**	**SD**	**Min**	**Median**	**Max**	**IQR**	**CV**
**Baseline**	-12.48	2.46	-25.84	-11.96	-8.22	2.87	-0.2
**HIA+ T1**	-13.81	3.06	-19.83	-14.5	-8.4	5.06	-0.22
**HIA+ T2**	-13.81	3.08	-21.42	-13.53	-7.96	4.33	-0.22
**HIA+ T3**	-12.59	2.47	-18.12	-12.05	-8.49	3.76	-0.2
**HIA- T1**	-12.07	2.31	-16.76	-11.71	-9.39	2.91	-0.19
**HIA- T2**	-12.87	2.17	-16.62	-13.06	-6.96	1.67	-0.17

**HIA- T3**	-12.95	2.11	-19.62	-12.63	-10.29	1.98	-0.16
**Uninjured T2**	-12.91	2.68	-18.88	-12.71	-4.96	3.04	-0.21
**Uninjured T3**	-13.13	2.57	-18.48	-12.52	-9.09	3.93	-0.2
**MSK T2**	-14.19	2.69	-20.59	-14.44	-9.39	3.41	-0.19
**MSK T3**	-13.18	2.32	-17.03	-13.57	-9.29	3.5	-0.18

tRNA18.ArgCCT	Mean	SD	Min	Median	Max	IQR	CV
**Baseline**	-22.9	1.62	-26.92	-23	-18.51	2.11	-0.07
**HIA+ T1**	-22.56	1.67	-26.42	-22.15	-19.59	2.02	-0.07
**HIA+ T2**	-22.31	1.8	-27.33	-22.1	-19.26	2.31	-0.08
**HIA+ T3**	-22.59	2.16	-26.36	-22.31	-18.11	3.5	-0.1
**HIA- T1**	-22.77	1.53	-25.76	-22.59	-19.4	1.86	-0.07
**HIA- T2**	-23.77	1.67	-26.61	-23.61	-21.31	2.86	-0.07
**HIA- T3**	-23.08	1.28	-25.24	-23.08	-20.22	1.35	-0.06
**Uninjured T2**	-22.8	1.66	-26.3	-22.84	-18.87	2.31	-0.07
**Uninjured T3**	-22.6	1.59	-26.05	-22.8	-18.72	2.23	-0.07
**MSK T2**	-23.57	1.45	-26.6	-23.45	-21.27	1.62	-0.06
**MSK T3**	-23.74	1.6	-26.49	-23.87	-21.02	2.52	-0.07

U6.1249	Mean	SD	Min	Median	Max	IQR	CV
**Baseline**	-8.96	2.18	-14.88	-8.64	-4.36	2.8	-0.24
**HIA+ T1**	-9.89	2.48	-16.57	-9.42	-4.99	2.99	-0.25
**HIA+ T2**	-9.68	2.9	-17.46	-8.78	-5.31	4.57	-0.3
**HIA+ T3**	-10.16	3.17	-17.81	-9.81	-5.57	5.42	-0.31
**HIA- T1**	-10.27	2.56	-15.78	-10.14	-5.48	2.61	-0.25
**HIA- T2**	-9.83	1.67	-12.61	-9.77	-7.11	2.2	-0.17
**HIA- T3**	-9.99	2.73	-17.29	-9.09	-6.39	1.72	-0.27
**Uninjured T2**	-9.92	2.65	-15.08	-9.75	-5.32	3.51	-0.27
**Uninjured T3**	-8.89	2.3	-16.74	-8.7	-4.66	2.36	-0.26
**MSK T2**	-9.94	2.36	-14.58	-9.81	-5.74	3.57	-0.24
**MSK T3**	-10.51	1.89	-13.23	-10.36	-7.27	2.38	-0.18

U6.168	Mean	SD	Min	Median	Max	IQR	CV

**Baseline**	-16.82	1.56	-20.2	-16.76	-11.76	1.96	-0.09
**HIA+ T1**	-17.07	1.38	-20.17	-16.69	-15.07	1.94	-0.08
**HIA+ T2**	-16.74	1.67	-21.16	-16.53	-12.15	2.39	-0.1
**HIA+ T3**	-16.78	1.57	-20.3	-16.41	-14.48	2.47	-0.09
**HIA- T1**	-17.01	1.46	-19.91	-17.03	-13.77	2.24	-0.09
**HIA- T2**	-17.46	1.49	-20.04	-17.06	-15.32	2.06	-0.09
**HIA- T3**	-17.37	1.43	-19.74	-17.02	-15.37	2.12	-0.08
**Uninjured T2**	-17.16	1.55	-20.01	-17.13	-13.29	2.08	-0.09
**Uninjured T3**	-17.04	1.64	-20.15	-16.98	-13.97	2.41	-0.1
**MSK T2**	-17.78	1.62	-20.69	-17.89	13.73	2.48	-0.09
**MSK T3**	-17.6	1.92	-20.67	-17.64	-13.8	2.96	-0.11

**U6.428**	**Mean**	**SD**	**Min**	**Median**	**Max**	**IQR**	**CV**
**Baseline**	-12.76	1.69	-16.74	-12.74	-7.8	1.92	-0.13
**HIA+ T1**	-12.69	1.51	-16.27	-12.45	-10.55	2.02	-0.12
**HIA+ T2**	-12.87	1.62	-16.69	-12.87	-9.62	1.88	-0.13
**HIA+ T3**	-13.1	1.53	-16.65	-12.82	-9.7	1.86	-0.12
**HIA- T1**	-12.9	1.49	-15.61	-12.8	-10.43	2.4	-0.12
**HIA- T2**	-13.27	1.8	-16.31	-13.14	-10.05	2.71	-0.14
**HIA- T3**	-13.05	1.49	-16.29	-12.72	-10.84	2.29	-0.11
**Uninjured T2**	-13.33	1.56	-16.43	-13.42	-9.05	1.73	-0.12
**Uninjured T3**	-13.27	1.39	-15.72	-13.09	-10.78	2.31	-0.11
**MSK T2**	-13.6	1.69	-17.3	-13.89	-10.43	2.16	-0.12
**MSK T3**	-14.26	1.59	-17.55	-13.93	-11.46	2.41	-0.11

**uc022cjg1**	**Mean**	**SD**	**Min**	**Median**	**Max**	**IQR**	**CV**
**Baseline**	-22.64	0.94	-25.76	-22.62	-20.14	1.12	-0.04
**HIA+ T1**	-22.36	0.89	-24.48	-19.54	-20.72	0.94	-0.04
**HIA+ T2**	-22.04	1.05	-24.32	-22.12	-19.54	1.21	-0.05
**HIA+ T3**	-22.57	1.16	-25.04	-22.69	-19.63	1.43	-0.05
**HIA- T1**	-22.18	0.82	-23.64	-22.15	-20.47	1.03	-0.04

**HIA- T2**	-22.36	1.17	-25.66	-22.45	-19.44	1	-0.05
**HIA- T3**	-22.82	0.9	-25.3	-22.68	-21.58	0.74	-0.04
**Uninjured T2**	-22.28	1	-24.09	-22.38	-19.96	1.35	-0.04
**Uninjured T3**	-22.32	1.03	-24.59	-22.22	-19.25	1.03	-0.05
**MSK T2**	-22.65	0.99	-25.68	-22.77	-21.01	1.05	-0.04
**MSK T3**	-23.09	1.29	-26.61	-23.09	-20.75	1.29	-0.06

**Y-RNA.255**	**Mean**	**SD**	**Min**	**Median**	**Max**	**IQR**	**CV**
**Baseline**	-24.11	1.65	-27.32	-24.21	-19.59	2.42	-0.07
**HIA+ T1**	-24.28	1.6	-27.77	-23.99	-20.99	1.96	-0.07
**HIA+ T2**	-23.71	1.77	-27.23	-23.88	-19.37	2.59	-0.07
**HIA+ T3**	-23.91	1.76	-27.77	-23.53	-20.53	2.81	-0.07
**HIA- T1**	-24.411	1.43	-26.97	-24.23	-21.67	2.43	-0.061
**HIA- T2**	-24.94	1.4	-27.74	-24.6	-22.62	2.25	-0.06
**HIA- T3**	-24.45	1.29	-26.59	-24.41	-21.87	1.9	-0.05
**Uninjured T2**	-24.44	1.65	-28.621	-24.65	-20.7	2.47	-0.071
**Uninjured T3**	-23.99	1.97	-27.59	-24.25	-17.77	2.54	-0.08
**MSK T2**	-24.77	1.67	-27.38	-24.86	-19.95	2.1	-0.07
**MSK T3**	-25.06	1.47	-27.8	-25.26	-21.53	2.26	-0.061

Uncensored data

After independent review of the incidents, 47 cases were excluded due to incomplete HIA documentation or failure to identify a clear mechanism of injury on the video footage. The analysis of the complete dataset including the excluded incidents does not show substantial differences from the censored data. The full uncensored dataset analysis is reported in the eTable 9. The overlap with the previous analysis is evidenced in grey cells.

eTable9: Analysis of uncensored data. Cells in grey match the biomarkers of eTable 3,4 and 5

**HIA+ (T2) vs Uninjured (T2)**	**t-test**<br>**p-value**
hsa-let-7a-5p	0.002
hsa-let-7f-5p	0.000
hsa-let-7i-5p	0.021
hsa-miR-103a-3p	0.023
hsa-miR-107	0.049
hsa-miR-135b-5p	0.001
hsa-miR-34b-3p	0.001
put-miR-742	0.028
RNU6-4	0.007
RNU6-45	0.025
RNU6-6	0.018
RNU6-7	0.018
RNU6-73	0.018
U6.375	0.045
U6.601	0.040
YRNA-255	0.040

**HIA+ (T3) vs Uninjured (T3)**	**t-test**<br>**p-value**
hsa-miR-144-3p	0.016

**HIA+ (T1) vs HIA- (T1)**	**t-test**<br>**p-value**
hsa-miR-103a-5p	0.038
hsa-miR-126-3p	0.049
hsa-miR-34b-3p	0.016

**HIA+ (T2) vs HIA- (T2)**	**t-test**<br>**p-value**
hsa-let-7a-5p	0.000
hsa-let-7f-5p	0.000
hsa-let-7i-5p	0.001

hsa-miR-103a-3p	0.001
hsa-miR-107	0.009
hsa-miR-135b-5p	0.000
hsa-miR-144-3p	0.025
hsa-miR-148a-3p	0.042
hsa-miR-34b-3p	0.007
hsa-miR-92a-3p	0.001
put-miR-6	0.006
put-miR-742	0.044
RNU6-4	0.000
RNU6-45	0.000
RNU6-6	0.000
RNU6-7	0.000
RNU6-73	0.000
SNORAD3B-2	0.022
tRNA120-AlaAGC	0.014
tRNA18-ArgCCT	0.001
tRNA27-MetCAT	0.022
tRNA73-ArgCCG	0.003
U2.3	0.025
U6.168	0.031
U6.375	0.000
U6.601	0.000
YRNA-255	0.001

**HIA+ (T3) vs HIA- (T3)**	**t-test**<br>**p-value**
hsa-let-7a-5p	0.042
hsa-let-7f-5p	0.000
hsa-let-7i-5p	0.001
hsa-miR-103a-3p	0.008
hsa-miR-107	0.027
hsa-miR-126-3p	0.039
hsa-miR-144-3p	0.010
hsa-miR-21-5p	0.004
hsa-miR-34b-3p	0.050

RNU6-4	0.004
RNU6-45	0.004
RNU6-6	0.005
RNU6-7	0.009
RNU6-73	0.008
U2.3	0.033
U6.375	0.034
U6.601	0.014

**HIA+ (T2) vs MSK (T2)**	**t-test p-value**
hsa-miR-143-3p	0.023
hsa-miR-16-1-3p	0.020
RNU6-4	0.012
RNU6-45	0.006
RNU6-6	0.015
RNU6-7	0.005
RNU6-73	0.011
SNORA57	0.048
tRNA120-AlaAGC	0.003
tRNA18-ArgCCT	0.004
U6.168	0.007
U6.375	0.016
U6.601	0.026
Uco22cjg1	0.011
YRNA-255	0.013

**HIA+ (T3) vs MSK (T3)**	**t-test p-value**
hsa-let7f-5p	0.005
hsa-miR-135b-5p	0.001
hsa-miR-144-3p	0.005
hsa-miR-16-1-3p	0.001
hsa-miR-21-5p	0.019
hsa-miR-425-5p	0.001
RNU4-6P	0.021
SNORA57	0.004

tRNA120-AlaAGC	0.015
tRNA18-ArgCCT	0.019
tRNA27-MetCAT	0.049
U6.428	0.003
put-miR-6	0.044
YRNA-255	0.004

eTable 10. Classifications of MSK Injuries by body part and injury type

Injury details were available for samples from 22 players. The remainder either did not have information available (players from the Championship competition) or had an injury resulting in less than 24 hours lost from full participation.

n=22	Number (%)
**Body Part**	
Shoulder	2 (9.10)
Forearm/Hand	4 (18.18)
Thoracic Spine	1 (4.55)
Abdomen/Trunk	2 (9.10)
Hip	1 (4.55)
Knee	1 (4.55)
Lower Leg	3 (13.64)
Foot/Ankle	8 (36.36)
**Injury Type**	
Ligament Sprain	11 (50)
Haematoma/Contusion	4 (18.18)
Fracture	3 (13.64)
Muscle Strain	1 (4.55)
Dislocation	1 (4.55)
Other	2 (9.10)

eFigure 1: Heat map of KEGG pathway analysis of differentially expressed microRNAs found in the comparison HA+ vs HIA- at T1.

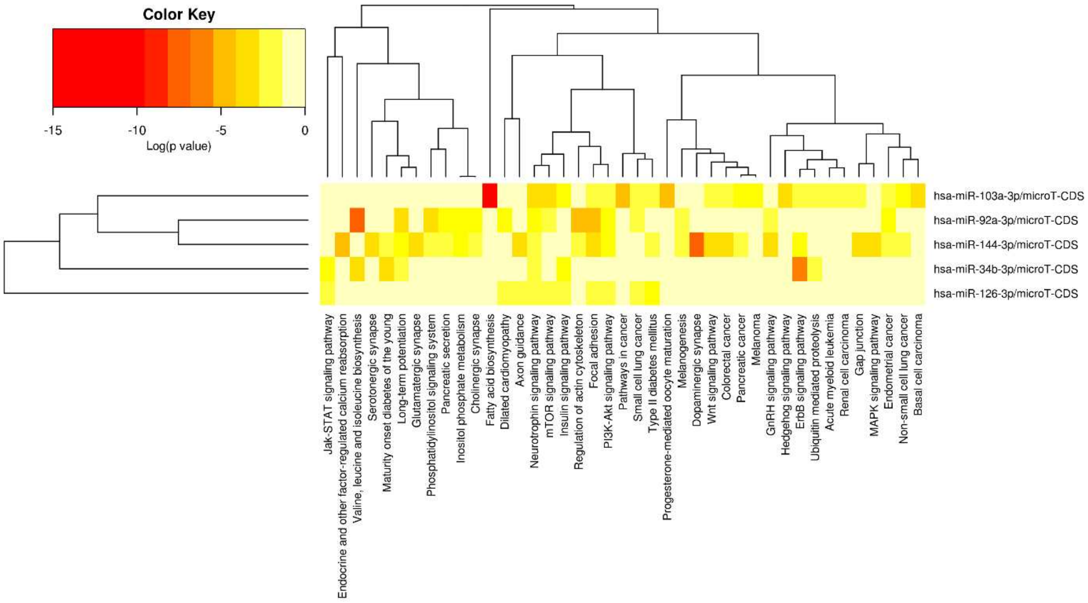

eFigure 2: Heat map of KEGG pathway analysis of differentially expressed microRNAs found in the comparison HA+ vs HIA- at T2.

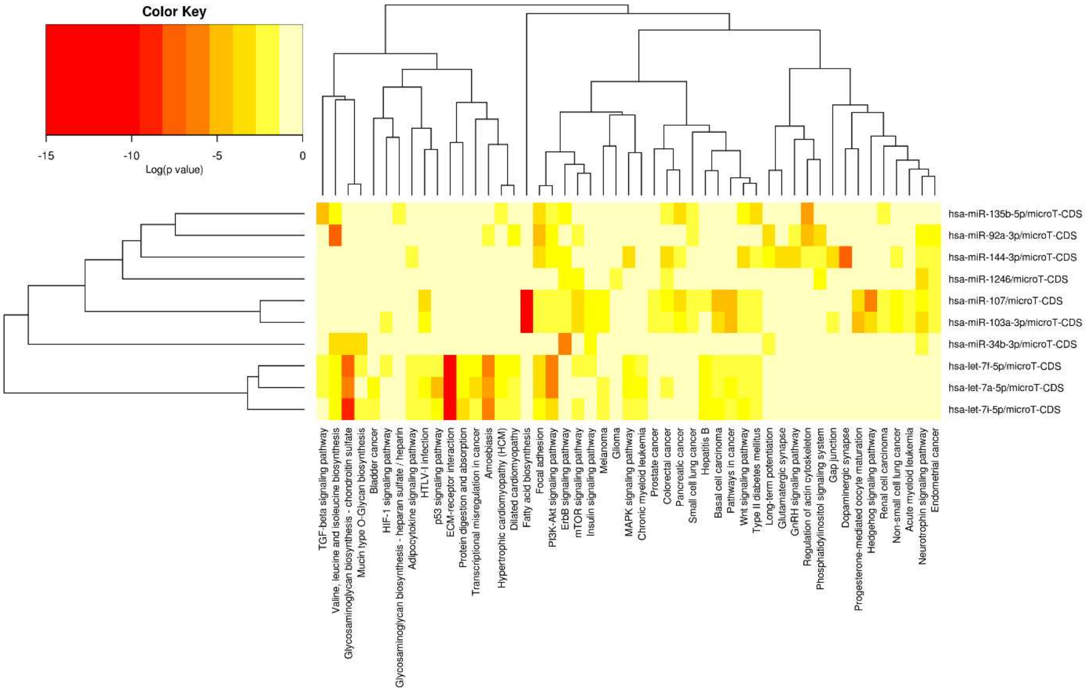

eFigure 3: Heat map of KEGG pathway analysis of differentially expressed microRNAs found in the comparison HA+ vs HIA- at T3.

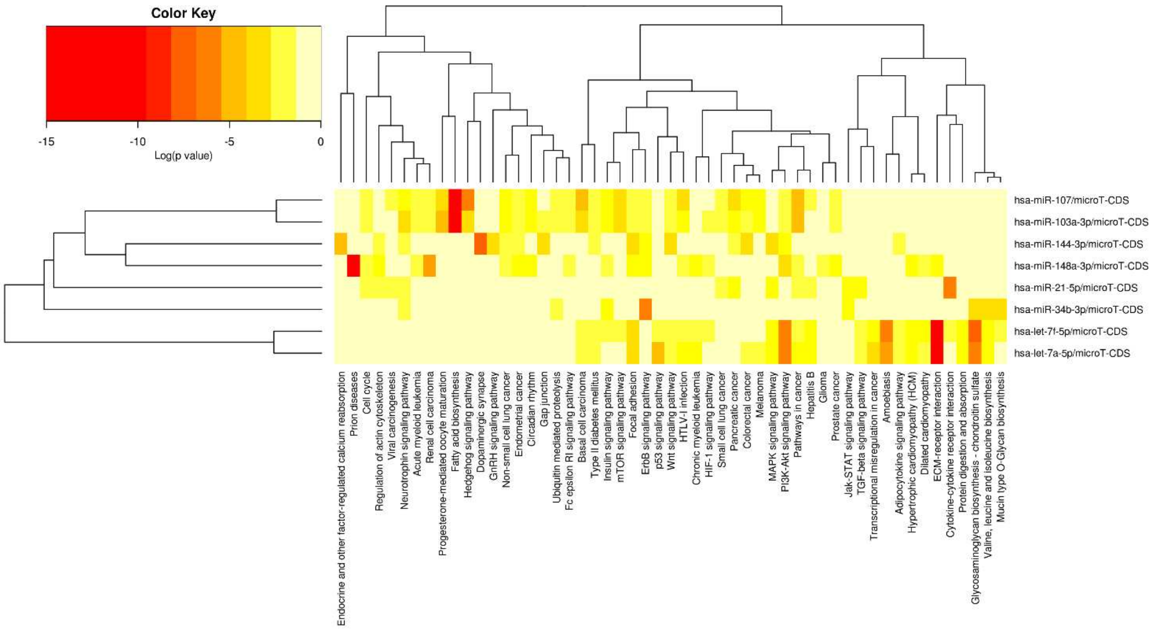

References

1 HIA_Protocol_Detailed_Description_Landscape_EN https://playerwelfare.worldrugby.org/content/getfile.php?h=030cc0e5445023d2887628100863ace2&p=downloads/concussion/HIA_Protocol_Detailed_Description_Landscape_EN.pdf.

2 Langmead B, Salzberg SL. Fast gapped-read alignment with Bowtie 2. *Nat Methods* 2012; 9(4): 357–9.

3 Wu Y, Wei B, Liu H, et al. MiRPara: a SVM-based software tool for prediction of most probable microRNA coding regions in genome scale sequences. *BMC Bioinformatics* 2011; 12: 107.

4 Kozomara A, Griffiths-Jones S. miRBase: integrating microRNA annotation and deep-sequencing data. *Nucleic Acids Res* 2011; 39: D152–7.

5 Griffiths-Jones S, Saini HK, van Dongen S, Enright AJ. miRBase: tools for microRNA genomics. *Nucleic Acids Res* 2008; 36: D154–8.

6 McCarthy DJ, Chen Y, Smyth GK. Differential expression analysis of multifactor RNA-Seq experiments with respect to biological variation. *Nucleic Acids Res* 2012; 40(10): 4288–97.

7 Andersen CL, Ledet-Jensen J, Ørntoft T**.** Normalization of real-time quantitative RT-PCR data: a model based variance estimation approach to identify genes suited for normalization - applied to bladder- and colon-cancer data-sets. *Cancer Research* 2004 (64): 5245-5250.
